# High‐Yield Convenient Mass Production of High‐Quality Homogenous Human Induced Pluripotent Stem Cell Spheroids under Rho‐Associated Kinase Inhibitor‐Free 3D Culture Enabled by Micropatterning and Cold‐Triggered Chemical‐Free Cell Detachment

**DOI:** 10.1002/smsc.202500201

**Published:** 2025-07-11

**Authors:** Zhiyuan Wang, Samantha Stewart, Mitsuo Kumagai, Ethan Wang, Wenquan Ou, Yongyu Lu, Taotao Meng, Cancan Xu, Yi Hong, Shenqiang Ren, Xiaoming He

**Affiliations:** ^1^ Fischell Department of Bioengineering University of Maryland, College Park Maryland 20742 USA; ^2^ Department of Materials Science and Engineering University of Maryland, College Park Maryland 20742 USA; ^3^ Department of Bioengineering University of Texas at Arlington Arlington, Texas 76019 USA; ^4^ Robert E. Fischell Institute for Biomedical Devices University of Maryland, College Park Maryland 20742 USA

**Keywords:** cold‐responsive, hiPSCs, microcontact printing, pluripotent stem cells, pNIPAAm, ROCK inhibitors

## Abstract

Human induced pluripotent stem cells (hiPSCs) show great promise for personalized cell‐based medicine, as they can be derived from easily accessible somatic cells and differentiated into all three germ layers without ethical concerns. This requires mass production of hiPSCs in 3D. However, contemporary methods for 3D culture result in hiPSC spheroids with significant size heterogeneity that is undesired for controlled differentiation and require the use of a high concentration of Rho‐associated kinase inhibitor (RI) to improve the cell viability. Unfortunately, a high concentration of RI causes uncontrolled spontaneous differentiation. To address these challenges, a cold‐responsive micropatterned dish (crMPD) is developed by spin‐coating a thin layer of cold‐responsive polymer on a cell culture dish and further microcontact printing cell attachment micropatterns on top of the coating layer. The hiPSCs attach and proliferate exclusively within the micropatterned areas to form a large number of uniform hiPSC colonies that can be detached as a whole by putting the crMPD on ice for ≈5–15 min. Under 3D culture without RI, the colonies can quickly self‐assemble into homogeneous hiPSC spheroids with high viability, yield, and pluripotency. This ingeniouscrMPD technology may be invaluable to facilitate widespread application of hiPSCs in research and personalized medicine.

## Graphical abstract

Cold‐responsive micropatterned dishes are fabricated by spin‐coating cell culture dishes with a cold‐responsive polymer and microcontact printing cell‐attachment micropatterns on top of the polymer. The micropatterns can be physically detached as a whole by ice‐cold cooling without using any chemical agents, enabling convenient large‐scale production of homogeneous 3D human induced pluripotent stem cell spheroids with high viability, yield, and quality.

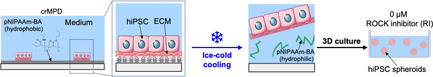

## 1 Introduction

Human induced pluripotent stem cells (hiPSCs), with their capability of differentiating into cell types of all three germ layers,^[^ ^1^ ^]^ hold tremendous potential for personalized cell‐based medicine.^[^ ^2^, ^3^ ^]^ The use of autologous hiPSCs compared to human embryonic stem cells (hESCs, usually not autologous) avoids concerns over immunoreactions and ethical issues.^[^ ^4^ ^]^ These hiPSCs have numerous applications for personalized modeling and treating disease, such as cell‐based therapy,^[^ ^5^, ^6^ ^]^ tissue and organ engineering,^[^ ^7^, ^8^, ^9^ ^]^ drug discovery,^[^ ^10^ ^]^ and toxicological screening.^[^ ^11^, ^12^ ^]^ For many of these applications, large‐scale production of the hiPSCs via 3D culture to form hiPSC spheroids is ideal. These 3D hiPSC spheroids mimic some structures of the developing embryo^[^ ^13^ ^]^ and are used to start lineage‐specific differentiation toward many different lineages like cardiac^[^ ^14^, ^15^ ^]^ and neural.^[^ ^16^, ^17^ ^]^ Cell differentiation outcomes using hiPSC spheroids depend greatly on the homogeneity and quality of the hiPSC spheroids.^[^ ^18^ ^]^ The size of the pluripotent cell spheroids also plays a large role in the effectiveness of differentiation to a specific lineage,^[^ ^15^, ^19^ ^]^ with spheroids that vary too greatly in size compromising the purity of directed differentiation.^[^ ^20^ ^]^ Spheroids of controllable and reproducible size are ideal for the best cell quality, viability, and directed differentiation into specific lineages.^[^ ^14^, ^21^ ^]^

Several methodologies have been developed with the goal of mass‐producing reliable and reproducible pluripotent spheroids, such as hanging drop/suspension culture,^[^ ^22^ ^]^ microwell arrays,^[^ ^21^, ^23^, ^24^ ^]^ microfluidics,^[^ ^25^ ^]^ and bioreactors.^[^ ^26^ ^]^ Although promising, these methods are often complex, labor‐intensive/time‐consuming, and/or expensive^[^ ^27^ ^]^ and have been used for the formation of spontaneously differentiated embryoid bodies instead of undifferentiated hiPSC spheroids.^[^ ^28^ ^]^ Two‐dimensional (2D) culture systems offer several advantages for hiPSC culture, namely for pluripotency maintenance and quality control.^[^ ^29^ ^]^ To create multicellular spheroids from 2D culture systems, hiPSC colonies must be detached from the 2D surface and allowed to self‐assemble into spheroids. Conventional detachment methods based on mechanical dissociation have seen some level of success with the development of technologies such as the StemPro EZPassage for 2D passaging. However, these methods can be laborious and dependent on the operator's experience or skills in selecting undifferentiated regions of hiPSC colonies as well as preventing excess mechanical damage to hiPSCs.^[^ ^30^ ^]^ Alternatively, enzymatic or chelating agents, such as the commercially available Versene, have been used but more often result in dissociation of the hiPSCs into single cells, which may quickly die of apoptosis and thereby result in poor spheroid yield.^[^ ^14^ ^]^ Protective agents like Rho‐associated kinase (ROCK) inhibitor (RI), Y‐27 632, can be added to suppress dissociation‐induced apoptosis of hiPSCs in suspension culture.^[^ ^31^ ^]^ However, adding RI is not ideal for hiPSC spheroid production for cell‐based medicine as it may cause spontaneous differentiation of hiPSCs toward the mesendoderm^[^ ^32^ ^]^ and neuroectoderm.^[^ ^33^ ^]^ Furthermore, RI has been shown to decrease pluripotency markers in 3D hiPSC spheroids and subsequently negatively impact their directed cardiac differentiation.^[^ ^14^ ^]^ Other methods force cell aggregation via centrifugation, which can potentially damage the cells and add more steps to 3D hiPSC spheroid protocols.^[^ ^34^ ^]^ Therefore, there is a need for better protocols to produce 3D hiPSC spheroids that are highly pluripotent and homogenous in size and do not require the use of RI.

Recently, there has been considerable interest in developing thermoresponsive cell culture surfaces as a method for enzyme‐free cell detachment.^[^ ^35^, ^36^, ^37^ ^]^ One of the most common polymers used for this application is poly(N‐isopropyl acrylamide) (pNIPAAm).^[^ ^38^ ^]^ This thermoresponsive polymer has the unique property of undergoing a phase/conformation transition when cooled below its lower critical solution temperature (LCST), altering the property of the polymer from a hydrophobic nature to a hydrophilic one.^[^ ^39^ ^]^ Reducing the temperature of the cell culture dish can thereby facilitate cell detachment without altering cell morphology or quality.^[^ ^37^ ^]^ However, many of the pNIPAAm copolymers used have cell detachment temperatures of ≈16–30 °C,^[^ ^37^, ^40^, ^41^ ^]^ which can make cell culture difficult, as they are close to room temperature (≈22 °C) and cell culture involves operations (e.g., changing medium and observing cells under microscope) at room temperature. Additionally, most studies evenly coated an extracellular matrix (ECM) component on top of the pNIPAAm copolymer for cell attachment to produce large cell sheets^[^ ^42^ ^]^ or detach hiPSC colonies of uncontrolled sizes.^[^ ^37^ ^]^ Microcontact printing is a simple technique that precisely and gently transfers a bioink like a protein solution from a stamp design to a surface.^[^ ^43^, ^44^ ^]^ Although this technique has been used to design cell attachment domains of uniform size to produce similarly sized 2D hiPSC and hESC colonies,^[^ ^45^, ^46^ ^]^ it has never been used to fabricate ink patterns on an ice‐cold‐responsive surface.

In this study, a pNIPAAm‐butyl acrylate (BA) copolymer is synthesized to have an LCST of 4–8 °C for surface coating to make a cold‐responsive surface for convenient ice‐cold‐triggered cell detachment. This cold‐responsive surface is then micropatterned with a design of Matrigel islands with controllable and uniform size to produce homogeneous hiPSC colonies. This cold‐responsive micropatterned dish (crMPD) can then be cooled on ice (≈0 °C) to gently release the homogeneous hiPSC colonies as a whole with negligible single hiPSCs in the absence of any enzymatic or chelating detachment agents. As a result, these hiPSCs are able to form hiPSC spheroids with high viability, high yield, high homogeneity, and high expression of pluripotency markers in the absence of RI. This novel crMPD‐based method is not only cost‐ and time‐effective compared to other hiPSC spheroid production methods but also produces high‐quality hiPSC spheroids with a homogeneous size, which are desirable characteristics for downstream differentiation of the hiPSCs into specific cell lineages, to facilitate the widespread use of hiPSCs for fundamental research and personalized cell‐based medicine.

## 2 Results

**Figure 1:**
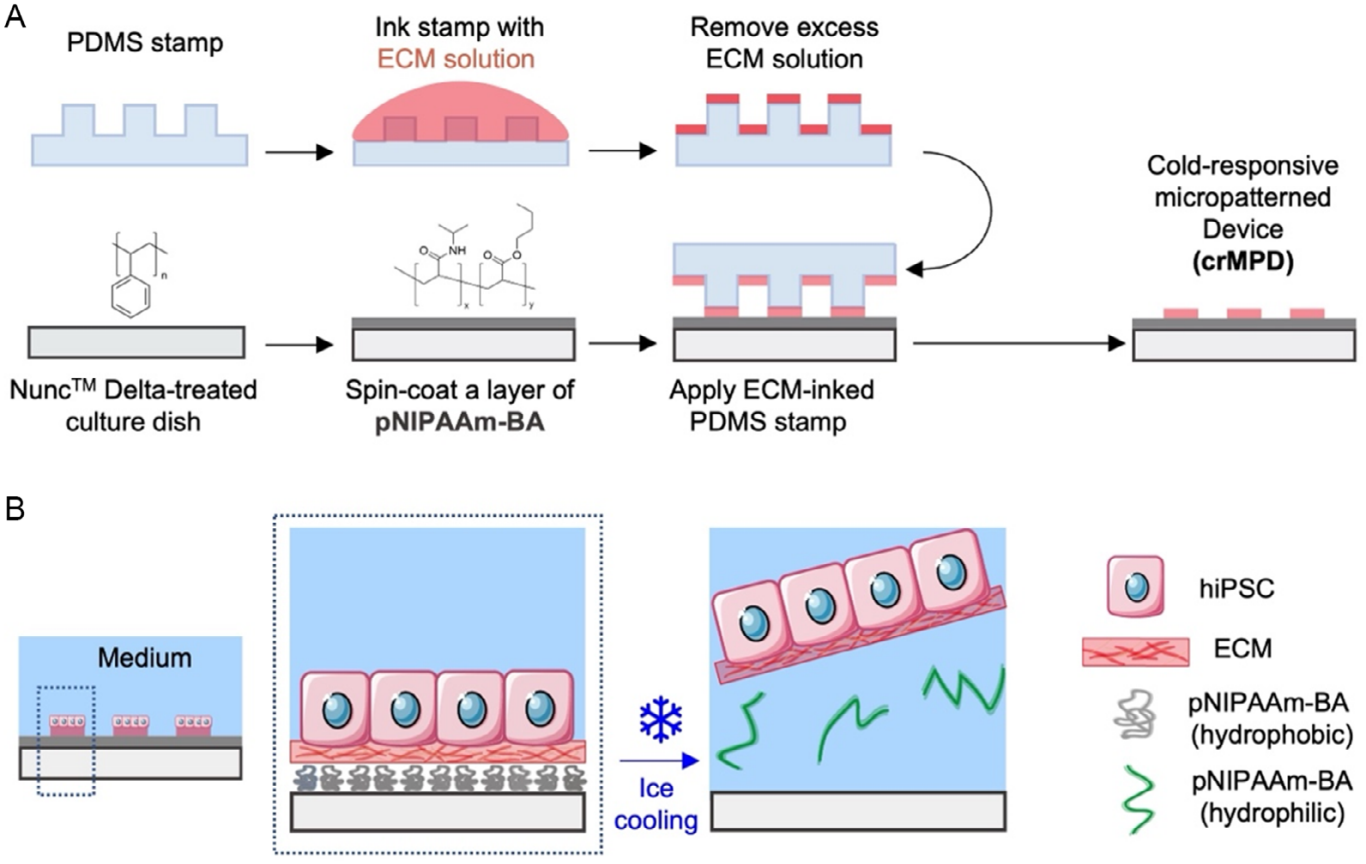
Schematic illustrations of the fabrication and use of the crMPD. A) A PDMS stamp prefabricated with the desired features for surface patterning with cell attachment areas (ECM) is inked with the ECM solution. Then, the excess ECM solution is removed via aspiration and blowing with N_2_. Concurrently, a Nunc Delta‐treated polystyrene dish is spin coated with a layer of cold‐responsive polymer, pNIPAAm‐BA (x:y = 5.8, *x* and *y* are the molar amounts of NIPAAm and BA, respectively), to create a cold‐responsive surface. The inked PDMS stamp is then placed pattern side down onto the cold‐responsive surface to pattern the surface with ECM for cell attachment, resulting in the final product, the crMPD. The ECM pattern used in this study is circular islands of Matrigel for hiPSC attachment. B) hiPSCs can attach on the crMPD only where there are the Matrigel islands created by the micropatterning with the PDMS stamp. At 37 °C, the pNIPAAm‐BA is insoluble in water/medium, and the polymer coating on the dish remains intact. Upon ice cooling (≈0 °C) below the LCST of the polymer (4–8 °C), the pNIPAAm‐BA becomes more hydrophilic and dissolves in the culture medium, releasing the hiPSCs as a whole cluster/colony from the surface.

This study demonstrates the fabrication (**Figure** **1**A) and use (Figure 1B) of a crMPD for convenient mass production of homogenous hiPSC spheroids with high viability and pluripotency without the use of any RI during 3D culture. To fabricate the crMPD (Figure 1A), a PDMS stamp is first made via photolithography and soft lithography with the desired micropatterns or islands. This stamp is inked with ECM solution (20 μg mL^−1^ Matrigel here) to allow the proteins for cell attachment to adsorb onto the micropatterns of the PDMS stamp. Simultaneously, the Nunc Delta‐treated polystyrene dish is spin‐coated with a cold‐responsive polymer, pNIPAAm‐BA. After excess ECM solution is aspirated and blown off the PDMS stamp with nitrogen gas, the inked stamp is placed feature‐side down on the pNIPAAm‐BA‐coated surface to microcontact print the cold‐responsive surface with ECM micropatterns where the cells can attach, creating the crMPD. Cells can then be seeded in the crMPD, where they attach only on the ECM pattern (Figure 1B). To release the cells from the ECM micropatterns in the crMPD, the dish is placed on ice (≈0 °C) to cool the pNIPAAm‐BA coating below its LCST (4–8 °C). As the pNIPAAm‐BA is cooled below its LCST, the polymer undergoes a phase change to become hydrophilic and water soluble, whereby it dissolves in the medium and releases the cell colony as a whole from the dish without the need of any enzyme or chelating agent to detach them from the surface (Figure 1B).

### 2.1 Characterization of crMPD

**Figure 2:**
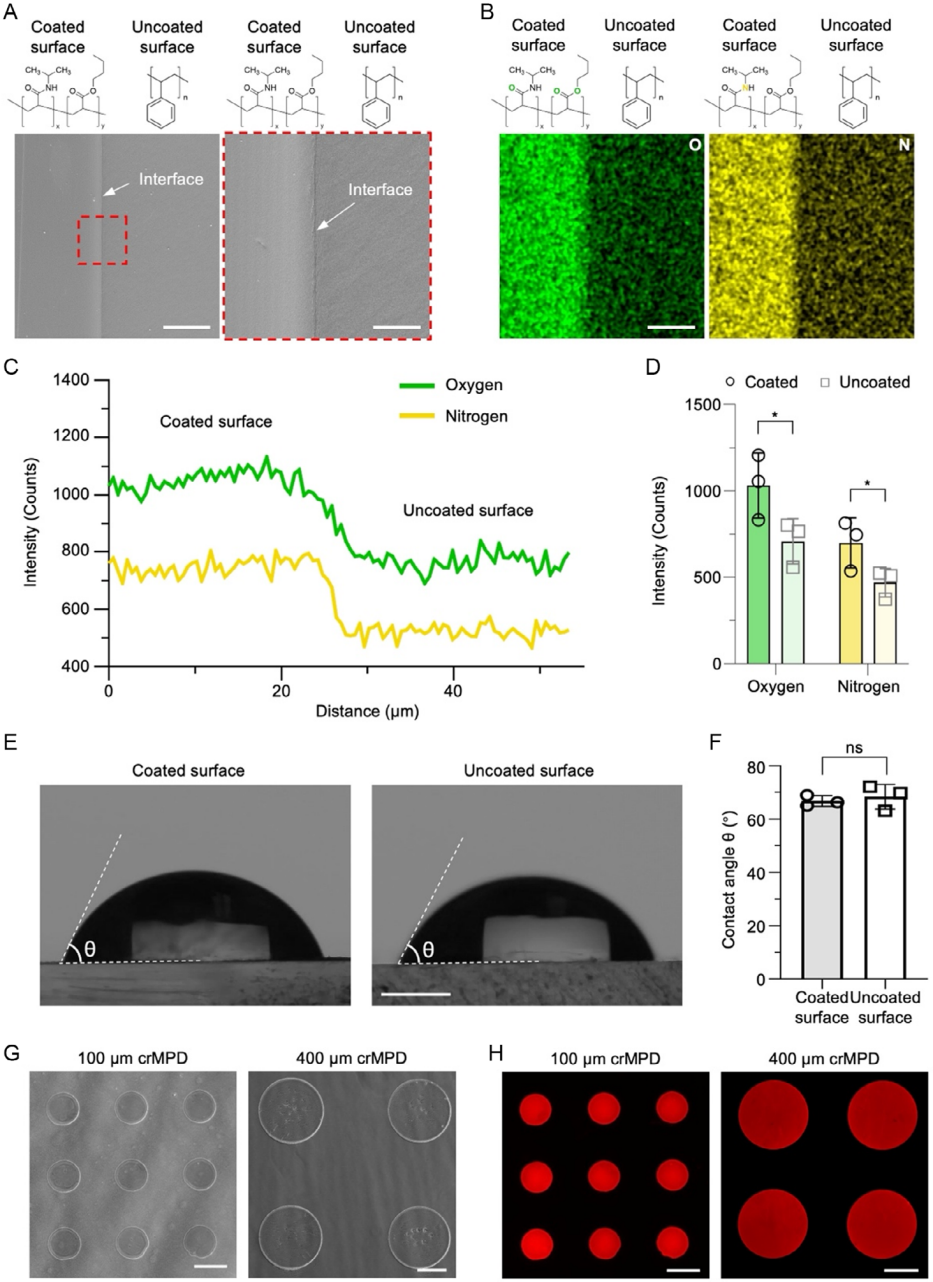
Characterization of the crMPD. A) Representative SEM image (left) together with a zoom‐in view (right) showing the interface of the uncoated Nunc Delta‐treated polystyrene surface and pNIPAAm‐BA‐coated surfaces. Scale bars: 50 μm (left) and 10 μm (right). B) EDXS mapping of oxygen (left) and nitrogen (right) in the surface layer of the uncoated versus coated surface. The brightness represents the intensity of the element's signal. The weak signals of nitrogen and oxygen on the uncoated side are due to residual oxygen and nitrogen gases in the vacuum of the sample chamber or the Nunc Delta treatment to enhance cell adhesion. Scale bar: 10 μm. C) EDXS line scan from the coated to uncoated surface. The intensity of both oxygen and nitrogen on the coated surface is higher than that on the uncoated surface. D) Comparison of the average intensity of oxygen and nitrogen from the EDXS line scans of the coated surface versus uncoated surface. E) Representative images of the water droplet on uncoated Nunc Delta‐treated polystyrene surface (left) and pNIPAAm‐BA‐coated surface (right) showing the contact angle (*θ*). Scale bar: 100 μm. F) Quantitative data of contact angles of water droplets on the uncoated versus pNIPAAm‐BA‐coated surface. The contact angle of uncoated Nunc Delta‐treated polystyrene surface is smaller than the uncoated polystyrene surface reported in the literature because the Nunc Delta surface treatment enhances the hydrophilicity to facilitate cell adhesion. G) Phase images of the micropatterned ECM (Matrigel) islands of different sizes (left: 100 μm, right: 400 μm) on the cold‐responsive pNIPAM‐BA layer in the crMPD. Both 100 and 400 μm Matrigel islands are uniform and round. Scale bars: 100 μm (left) and 200 μm (right). H) Fluorescence images of the micropatterns made with a mixture of Matrigel and Rhodamine B as the ink. Left, 100 μm micropatterns. Right, 400 μm micropatterns. The fluorescence images show uniform distribution of Rhodamine B (with red fluorescence) in the micropatterns. Scale bar: 100 μm (left) and 200 μm (right). The *x*:*y* ratio of all pNIPAAm‐BA copolymers used in this study (*x* for NIPAAm and y for BA) is 5.8. Statistical analyses were done using two‐tailed paired *t*‐test D) and two‐tailed unpaired *t*‐test F). *p < 0.05, and ns represents not significant. *n* = 3 independent runs. Error bars represent standard deviation.

Representative images of the crMPDs with Matrigel islands of both 100 and 400 μm (in diameter) fabricated using the aforementioned method (Figure 1A) are shown in Figure S1, Supporting Information. Successful coating of pNIPAAm‐BA on the surface of the polystyrene cell culture dish is confirmed by scanning electron microscopy (SEM) image (**Figure** **2**A) of the area with an interface between the uncoated and the pNIPAAm‐BA‐coated surface. The successful coating is further confirmed by energy dispersive X‐ray spectroscopy (EDXS) analysis of nitrogen (N) and oxygen (O) elements in the coated versus uncoated surface layers. As shown in Figure 2B–D, the pNIPAAm‐BA‐coated surface has a significantly higher occurrence of both N and O elements than the uncoated surface, because the pNIPAAm‐BA polymer in the coated surface contains both N and O, while the polystyrene polymer in the uncoated surface does not. The weak signals of nitrogen and oxygen on the uncoated side are due to residual oxygen and nitrogen gases in the sample chamber of EDXS and the Nunc Delta treatment. Contact angle analysis (Figure 2E–F) indicates that the hydrophilicity (or hydrophobicity) of the coated surface is not significantly different from the uncoated Nunc Delta‐treated polystyrene surface. It is worth noting that the contact angle of the uncoated Nunc Delta‐treated surface (≈65°, Figure 2F) is smaller than the contact angle of the untreated polystyrene surface (≈90° in literature),^[^ ^47^, ^48^ ^]^ indicating the Nunc Delta treatment makes the polystyrene surface more hydrophilic. Microscopic phase images of the dishes further confirm that the Matrigel islands are successfully patterned onto the pNIPAAm‐BA surface in the dishes with good homogeneity at the designed size (≈100 μm in diameter for the left panel in Figure 2G and ≈400 μm in diameter for the right panel in Figure 2G). To confirm the homogeneity of ECM materials inside each island, a mixture of fluorescent Rhodamine B and Matrigel was used as the ECM inking solution for making the micropatterns. Fluorescence images (Figure 2H) show that the microcontact printing method successfully patterned the pNIPAAm‐BA surface with the designed features (i.e., 100 or 400 μm Matrigel islands in diameter) from the PDMS stamps and with uniform distribution of Rhodamine B in the islands.

### 2.2 The crMPD for hiPSC Culture and Cold‐Triggered Release

**Figure 3:**
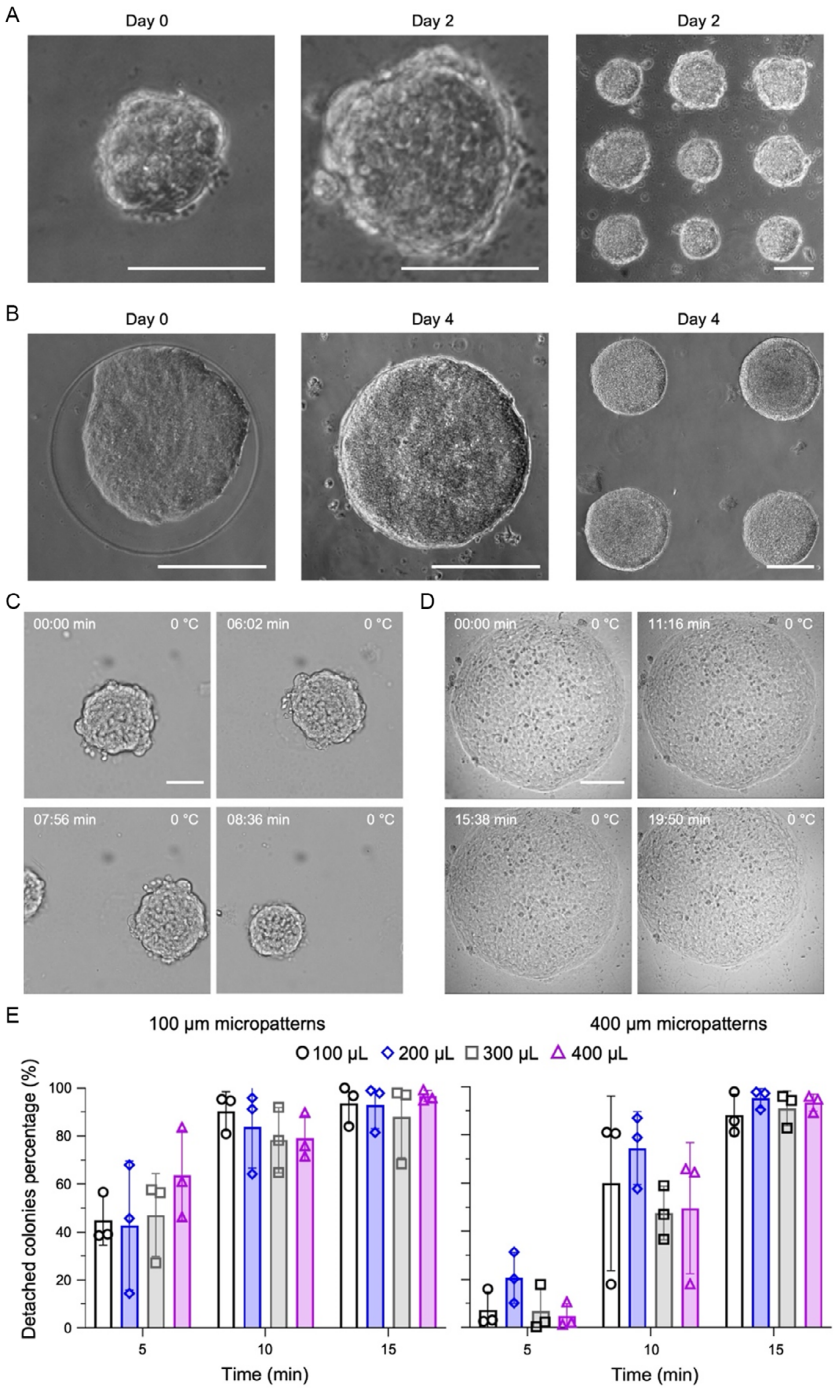
Culture and cold‐triggered release of hiPSCs in the crMPD. A–B) DF19‐9‐11 T.H hiPSC attachment on 100 μm (A) and 400 μm (B) Matrigel islands/micropatterns on crMPD. The hiPSCs could attach and grow in 2–4 days to form well‐defined colonies with clear boundary on both 100 and 400 μm Matrigel islands. Scale bars: 100 μm for panel A and 200 μm for panel B. C,D) Cryomicroscopy images showing detachment of Matrigel islands of different size C) 100 μm, D) 400 μm) from the crMPD upon cooling at 0 °C. Scale bars: 50 μm for panel C and 100 μm for panel D. E) Detaching percentage of hiPSC colonies grown in crMPDs with different coating thicknesses of pNIPAM‐BA at different time points of cooling at 0 °C. The 100, 200, 300, and 400 μL of pNIPAAm‐BA solution represent 5.7, 11.4, 17.0, and 22.7 μm of coating thickness. There is no statistical difference in the detaching percentage between the four different pNIPAM‐BA coating thicknesses for both the 100 μm (left) and 400 μm (right) micropatterns at same detaching time points. Good detachment percentage (≈90%) can be achieved for micropatterns of both sizes after cooling on ice for 15 mins. Statistical analyses were done using one‐way ANOVA with Tukey's multiple comparisons test and correction. *n* = 3 independent runs. Error bars represent standard deviation.

To confirm hiPSCs can attach, grow/proliferate, and then subsequently detach from the crMPD, DF19‐9‐11T.H cells were seeded on a crMPD with micropatterned Matrigel islands of 100 μm and 400 μm in diameter at a density of 1 × 10^4^ cells cm^−2^. After culturing overnight, hiPSCs can be seen to successfully attach (**Figure** **3**A–B, left column, day 0) and proliferate (i.e., the colony becomes bigger, Figure 3A–B, middle column, day 2 for 100 μm patterns and day 4 for 400 μm patterns) on the micropatterned Matrigel islands in the crMPD. The hiPSC growth and proliferation are confined only to the Matrigel islands micropatterned on the pNIPAAm‐BA, allowing for the creation of defined hiPSC patterns, which in this case are round colonies of hiPSCs as seen in the lower magnification image of the crMPD on day 2 for 100 μm patterns (Figure 3A, right column) and day 4 for 400 μm patterns (Figure 3B, right column). After 2 or 4 days of culture in the crMPD, hiPSCs maintain normal colony morphology (compact/tight cellular packing with defined and smooth colony boundaries) on the Matrigel‐coated areas of the crMPD. These experiments were repeated using recombinant vitronectin in place of Matrigel as a defined and xeno‐free ECM protein for making the crMPD with micropatterned islands of 400 μm diameter (Figure S2A, Supporting Information). The hiPSCs can be seen to similarly attach after culturing overnight and proliferate to form well‐defined colonies with clear boundaries over 4 days (Figure S2B, Supporting Information).

Next, cryomicroscopy was used to confirm that cooling below the LCST of pNIPAAm‐BA can result in gentle and easy detachment of the 2D hiPSC colonies from the crMPD. The DF19‐9‐11 T.H hiPSCs were seeded in a crMPD patterned with a round Matrigel island of 100 μm in diameter and cultured for 2 days, or 400 μm in diameter and cultured for 4 days, and then cooled on a cryomicroscope stage to 0 °C. The temperature of the sample was held at 0 °C, and images of the hiPSC colony grown on the round island attachment site were taken over either 10 min (for 100 μm Matrigel islands) or 20 min (for 400 μm Matrigel islands). For the 100 μm micropatterns (Movie S1, Supporting Information, and Figure 3C), the round ≈100 μm hiPSC 2D colony can be observed to attach to the Matrigel island at 00:00 min. After ≈6 min of cooling at 0 °C, the hiPSC colony begins to detach from the round island, as it moves slightly away to the top and right from its original position in the image. The hiPSC colony continues to detach or move away from its original position in the crMPD until it leaves the imaging region completely by 08:36 min, when another hiPSC colony that has detached from a different round island attachment site enters the imaging region. For the 400 μm micropatterns (Movie S2, Supporting Information, and Figure 3D), the round ≈400 μm hiPSC 2D colony can be observed to attach on the Matrigel island at 00:00 min. After ≈11 min of cooling at 0 °C, the hiPSC colony begins to detach from the round island, as it moves slightly away to the top and left from its original position in the image. The 400 μm hiPSC colony continues to detach or move away from its original position in the crMPD until ≈20:00 at the end of the video recording. Compared to the 100 μm hiPSC colony, it exhibits smaller movements due to its much (≈4 times in diameter and 16 times in area) larger size and weight.

To further assess the impact of pNIPAM‐BA coating thickness on detachment efficiency, four different volumes of pNIPAM‐BA (in ethanol) solutions were used to spin coat pNIPAM‐BA on the 35 mm cell culture dish, resulting in crMPDs with four different thicknesses of the pNIPAM‐BA coating layer. Both 100 and 400 μm (diameter) micropatterns were microcontact printed on the resultant crMPDs. Then, DF19‐9‐11 T.H hiPSCs were seeded and cultured in the crMPDs for either 2 days (100 μm Matrigel islands) or 4 days (400 μm Matrigel islands). The percentage of detached hiPSC colonies from the crMPDs coated with different pNIPAM‐BA thicknesses was quantified after 5, 10, and 15 min of cooling on ice, and the results are shown in Figure 3E. There is no statistical difference between the different coating thicknesses of pNIPAM‐BA for both 100 μm (Figure 3E, left) and 400 μm (Figure 3E, right) crMPDs at all time points. Both 100 μm (Figure 3E, left) and 400 μm (Figure 3E, right) crMPD show good (≈90%) detachment percentage after cooling on ice for 15 min, although the detachment percentage appears higher for the smaller micropatterns at 10 min.

### 2.3 High Viability and Yield of hiPSCs Cold‐Detached from crMPD in 3D Culture without RI

The viability of hiPSCs detached from the crMPD with 400 μm (in diameter) Matrigel islands was investigated using a live/dead assay assessed by calcein AM (live/green) and propidium iodide (PI, dead/red) staining with three different hiPSC cell lines: DF19‐9‐11 T.H and IMR90‐1 purchased from WiCell, and a home‐made hiPSC cell line reprogrammed from human primary dermal fibroblast (HPF) using a commercial reprogramming kit. The hiPSCs were detached using either Versene or cold via the crMPD, then cultured in 3D for either 2 or 48 h before staining. The hiPSCs detached using Versene were supplemented with 0, 1, or 10 μm RI for 3D culture, while the hiPSCs detached from the crMPD were not supplemented with any RI.

**Figure 4:**
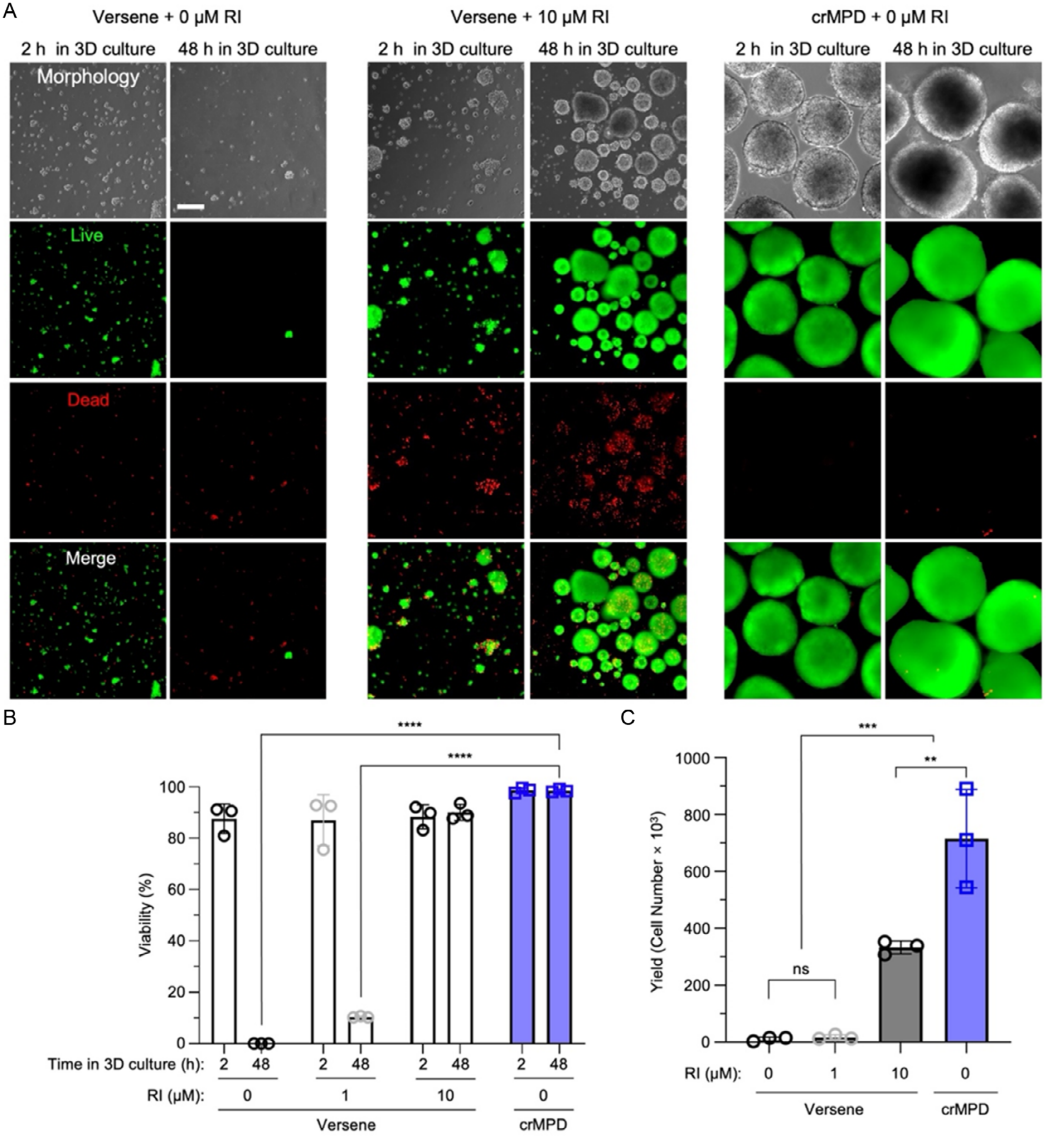
Viability and yield of hiPSCs postdetachment from the crMPD and under 3D suspension culture. A) Representative images and B) Quantitative data for the viability assessed by live/dead staining of DF19‐9‐11 T.H hiPSCs after 2 and 48 h of 3D suspension culture postdetachment using either Versene and supplementation with 0, 1, and 10 μm RI or cold treatment of crMPD with 400 μm‐diameter Matrigel islands and supplementation with 0 μm RI. C) Cell yield after 48 h of 3D suspension culture of the hiPSCs postdetachment under the aforementioned conditions. Statistical analyses were done using one‐way ANOVA with Tukey's multiple comparisons test and correction. **p < 0.01, ***p < 0.001, ****p < 0.0001, ns represents not significantly different. *n* = 3 independent runs. Scale bar: 200 μm. Error bars represent standard deviation.

Representative images of the DF19‐9‐11 T.H hiPSCs at 2 and 48 h postdetachment and in 3D culture are shown in **Figure** **4**A, with the quantitative viability data being shown in Figure 4B. After 2 h of 3D culture, the hiPSCs from all conditions show high viability (>85%). However, without RI supplementation, most of the hiPSCs detached using Versene die by 48 h in 3D culture (Versene + 0 μm RI, 0.3 ± 0.2% viability), even though most of the cells are alive after 2 h in 3D culture (87.6 ± 5.7%). Under this condition, although some cell aggregates could be seen in 3D culture at 2 h, most of them dissociate into single cells by 48 h. The supplementation of RI helps cells detached with Versene to survive 3D culture, as expected. Supplementation with a small amount of RI (1 μm) prevents 10.4 ± 0.3% hiPSCs from death at 48 h of 3D culture, while increasing the amount of RI to 10 μm RI results in a cell viability of 90.1 ± 3.1% at 48 h of 3D culture. Importantly, hiPSCs detached with cold treatment from the crMPD show high viability both at 2 h (98.7 ± 1.0%) and 48 h (98.5 ± 0.6%) postdetachment and 3D culture without the need for any RI. The hiPSCs detached from the crMPD show statistically significant higher viability than those from the Versene + 0 μm RI and Versene + 1 μm RI groups and similar (but slightly higher) viability to the Versene + 10 μm RI group at 48 h. Although both hiPSCs detached using Versene and supplemented with 10 μm RI and hiPSCs detached from the crMPD without RI are seen to mostly aggregate without many single cells after 2 h of 3D culture that grow into larger spheroids by 48 h, more dead single cells can be seen for the Versene‐detached cells even in the presence of 10 μm RI during 3D culture. A similar trend of the cell viability at 2 and 48 h is observable for two other hiPSC cell lines: IMR90‐1 cells (Figure S3 and S4, Supporting Information) and the reprogrammed hiPSCs (Figure S5 and S6, Supporting Information), which further confirms the crMPD can be used with cold treatment for cell detachment to improve hiPSC viability during 3D culture, independent of hiPSC types.

To determine the yield of hiPSCs from the aforementioned four different combinations of cell detachment methods and RI concentrations for 3D culture, the same number of hiPSCs were cultured in 3D suspension for 2 days, the resultant hiPSC spheroids were collected, and the spheroids were dissociated into single cells to count the cell number. The quantitative data on the yield of DF19‐9‐11 T.H are shown in Figure 4C. The hiPSC yield for the condition of cell detachment using Versene without any RI supplementation for 3D culture is negligible, as few cells survive to form spheroids in 3D culture without RI (Figure 4A–B). The addition of 1 μm RI to the Versene‐detached cells for 3D culture results in a slight but not statistically significant increase of the cell yield by ≈1.5 fold compared to the condition of Versene detachment without RI supplementation. Increasing the concentration of RI to 10 μm greatly and significantly enhances cell yield for Versene‐detached cells by ≈32 and ≈20 folds, compared to Versene‐detached cells with the 0 and 1 μm RI supplementation, respectively. Importantly, hiPSCs detached by ice‐cold treatment from the crMPD without any RI supplementation for 3D culture also show a greatly and significantly enhanced (by ≈2.1 fold) cell yield compared to the hiPSCs detached using Versene and supplemented with 10 μm RI for 3D culture. A similar trend in the cell yield for the four different conditions is also observable for the IMR90‐1 cells (Figure S7, Supporting Information) and the reprogrammed hiPSCs (Figure S8, Supporting Information), showing the greatly and significantly enhanced cell yield by using the crMPD with ice‐cold‐triggered detachment of hiPSCs for 3D culture is general for all hiPSCs.

### 2.4 High Homogeneity in Size and Roundness in Shape of hiPSC Spheroids Produced via crMPD

**Figure 5:**
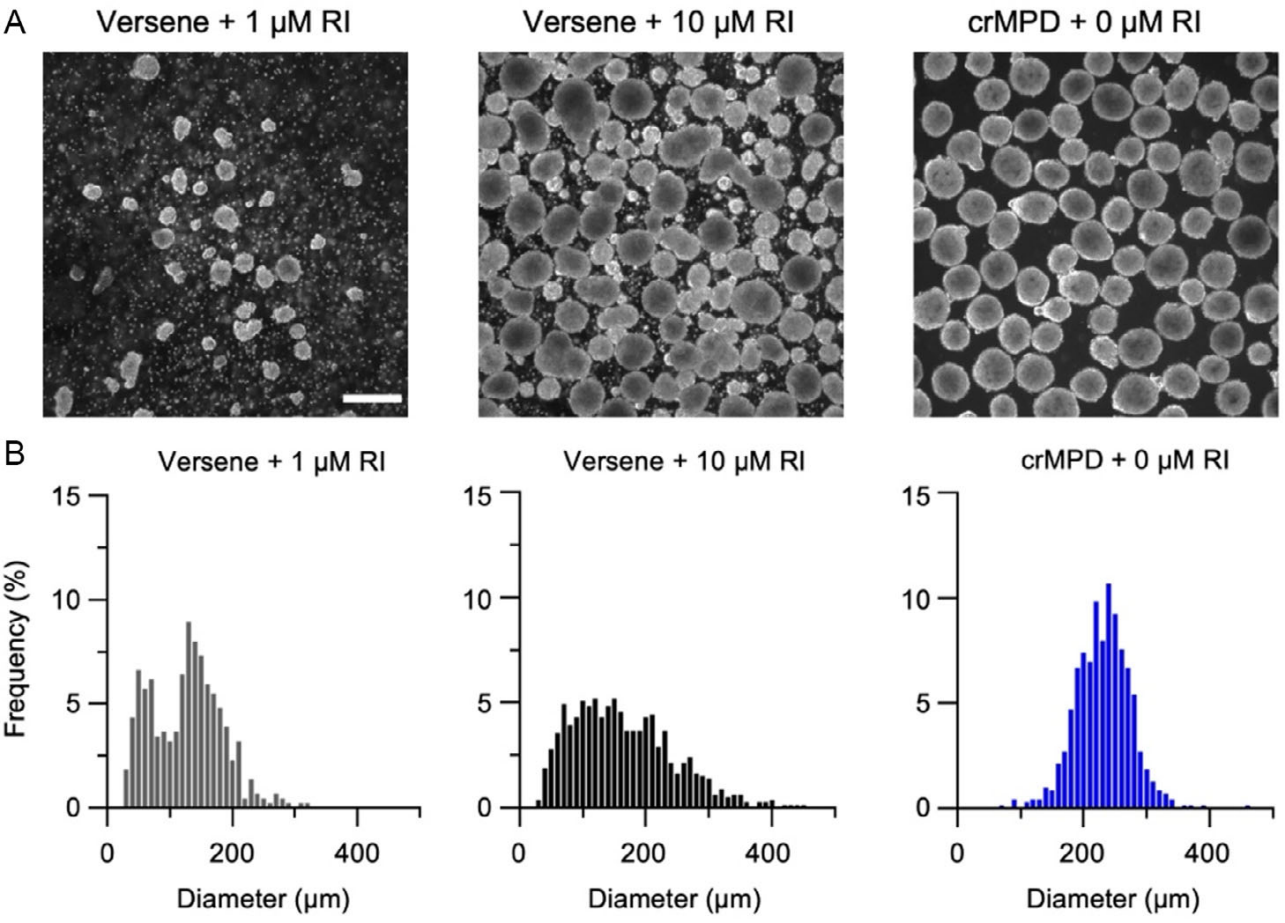
Homogeneous size of hiPSC spheroids produced by using the crMPD. A) Representative images, and B) histogram showing size distribution, of DF19‐9‐11 T.H hiPSC spheroids produced after detachment using either Versene supplemented with RI (1 or 10 μm) or cold treatment of the crMPD supplemented with 0 μm RI. *n* = 3 independent runs. Scale bar: 500 μm.

The size distributions of the hiPSC spheroids produced either using Versene with either 1 or 10 μm RI supplementation for 3D culture or by ice‐cold‐triggered detachment from crMPD with no RI supplementation for 3D culture were investigated to confirm the production of homogenous spheroids ideal for further differentiation of the cells. Both the hiPSCs detached using Versene with either 1 or 10 μm RI supplementation or from crMPD and the hiPSCs detached from crMPD with no RI supplementation were cultured in 3D suspension for 2 days. For all three different hiPSC lines, the spheroids produced using Versene detachment with 1 and 10 μm RI supplementation for 3D culture are similar in size and significantly smaller than the hiPSC spheroids made using the crMPD with cold‐triggered detachment and without any RI for 3D culture (Figure S9, Supporting Information). Furthermore, the distribution of the spheroid diameter from the three conditions differs greatly. Representative images of the DF19‐9‐11 T.H hiPSC spheroids produced from Versene‐detached hiPSCs supplemented with 1 or 10 μm IR for 3D culture and cold‐detached hiPSCs from crMPD without RI supplementation for 3D culture are shown in **Figure** **5**A. The hiPSC spheroids formed by using Versene detachment vary greatly in size due to uncontrolled colony size, as evidenced by the representative images and the accompanying wide size distributions shown in the histograms in Figure 5B. Importantly, the hiPSC spheroids generated with the crMPD are round and very homogeneous in size with a narrow Gaussian distribution, as seen in Figure 5A–B. A similar trend in the hiPSC spheroid homogeneity is also observable for the IMR90‐1 cells (Figure S10, Supporting Information) and the reprogrammed hiPSCs (Figure S11, Supporting Information), showing the improved homogeneity of the resultant 3D spheroids by using the crMPD with ice‐cold‐triggered detachment of hiPSCs for 3D culture is general for all hiPSCs. To study how the crMPD method may affect the roundness of hiPSC spheroids, circularity of the DF19‐9‐11 T.H, IMR90‐1, and reprogrammed hiPSC spheroids generated from Versene detachment followed by either 1 or 10 μm RI supplementation or from crMPD was quantified. Although there is no significant difference in circularity between the DF19‐9‐11 T.H hiPSC spheroids generated from the crMPD and Versene‐based methods (Figure S12A, Supporting Information), the crMPD can significantly improve the circularity or roundness of both IMR90‐1 (Figure S12B, Supporting Information) and reprogrammed hiPSC (Figure S12C) spheroids compared with the culture method of Versene with 1 μm RI. This shows the impact of the crMPD technology on the shape of 3D spheroids is cell‐type dependent.

### 2.5 High Pluripotency of hiPSC Spheroids Produced via crMPD with Cold‐Triggered Cell Detachment

**Figure 6:**
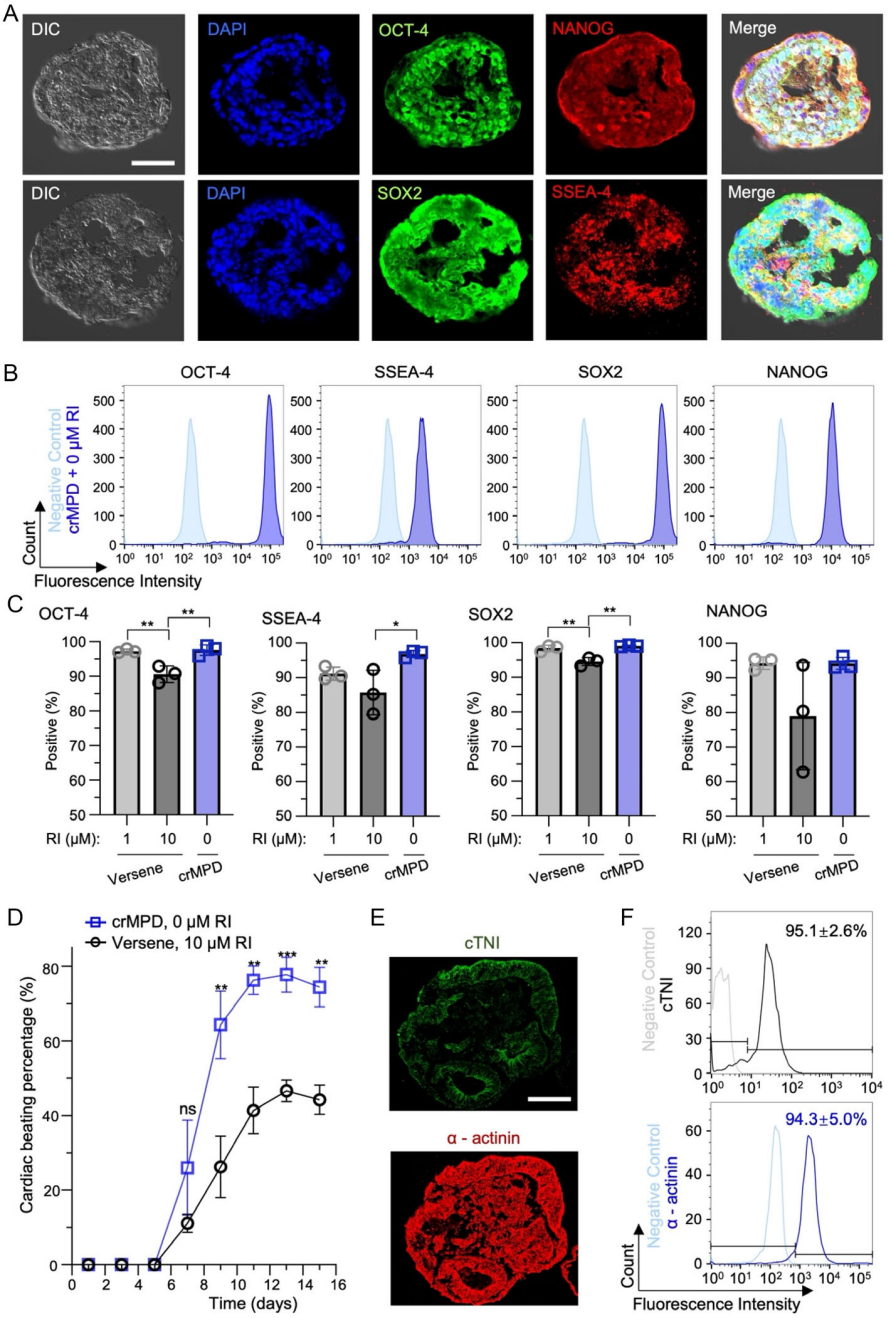
High pluripotency of hiPSCs produced using the crMPD. A) Representative images of DF19‐9‐11 T.H hiPSC spheroids produced using the crMPD, showing their high expression of pluripotency markers OCT‐4, NANOG, SOX2, and SSEA‐4 via immunostaining. DIC: differential interference contrast. Scale bar: 50 μm. B) Representative flow cytometry peaks of the pluripotency markers OCT‐4, SSEA4, SOX2, and NANOG of cells from hiPSC spheroids produced by cold detachment from crMPD and supplementation with 0 μm RI for 3D culture of the DF19‐9‐11 T.H hiPSCs. The negative controls (secondary antibody only, no primary antibody) are shown in light blue. C) Quantitative data from flow cytometry analysis showing expression of pluripotency markers OCT‐4, SSEA4, SOX2, and NANOG of cells in hiPSC spheroids produced either by Versene detachment supplemented with 1 or 10 μm RI for 3D culture or by cold detachment from crMPD and supplementation with 0 μm RI for 3D culture. D) Beating percentage of cardiac organoids produced from hiPSC spheroids grown from hiPSCs obtained via either Versene detachment with 10 μm RI for 3D culture or cold detachment from crMPD with 0 μm RI for 3D culture. E) Representative images of cardiac organoids (on day 15 of differentiation) differentiated from DF19‐9‐11 T.H hiPSC spheroids produced by the crMPD‐based method, showing their high expression of cardiac markers cTNI and α‐actinin. Scale bar: 250 μm. F) Representative flow cytometry peaks of cardiac markers cTNI and α‐actinin of cells in cardiac organoids (on day 15 of differentiation) differentiated from DF19‐9‐11 T.H hiPSC spheroids produced by the crMPD‐based method. The negative controls (secondary antibody only, no primary antibody) are shown in gray and light blue for the cTNI and α‐actinin data, respectively. Statistical analyses were done using one‐way ANOVA with Tukey's multiple comparisons test and correction for panel C and two‐tailed unpaired t‐test for panel E. *p < 0.05, **p < 0.01, ***p < 0.001, and ns: not significant. *n* = 3 independent runs. Error bars represent standard deviation.

To evaluate the quality of hiPSC spheroids produced using the crMPD, the expression of four pluripotency markers (OCT‐4, SSEA‐4, NANOG, SOX2) was investigated. Immunostaining shows that DF19‐9‐11 T.H hiPSC spheroids produced using the crMPD‐based method are highly positive for OCT‐4, SSEA‐4, NANOG, and SOX2 (**Figure** **6**A). Flow cytometry analyses were used to quantitatively evaluate the expression of the four pluripotency markers of hiPSC spheroids produced using the crMPD without RI supplementation compared to hiPSC spheroids produced using Versene detachment and supplementation with 1 or 10 μm RI. Representative peaks of pluripotency markers from all three conditions are given in Figure 6B and S13, Supporting Information. Although little overlap can be seen between the isotype control and the experimental fluorescence intensity peaks of all four pluripotency markers for all three different methods, the experimental fluorescence intensity peaks for the crMPD group are narrower and with clearer separation from the corresponding isotype peaks (Figure 6B) than the fluorescence intensity peaks for the Versene detachment groups with either 1 or 10 μm RI supplementation (Figure S13, Supporting Information), suggesting better quality of the hiPSCs obtained with the crMPD‐based approach than the two conventional methods. This is further confirmed by the quantitative data shown in Figure 6C for the percentage of hiPSCs that are positive for the four markers. The hiPSCs from the crMPD group are >95% positive for all four pluripotency markers with little variation. The expression of all four pluripotency markers is higher in hiPSCs from the crMPD group than in the Versene detachment method with 10 μm RI, and the difference is significant for OCT‐4, SSEA‐4, and SOX2. The same trend is observable when comparing the hiPSC spheroids produced by Versene detachment with 1 μm RI to 10 μm RI. Although there is no significant difference between the expression of pluripotency markers for the 1 μm RI Versene‐detached hiPSC spheroids and the 0 μm RI crMPD‐produced spheroids, the viability, yield, and homogeneity of the hiPSCs from the crMPD group are much better than the hiPSCs obtained by Versene detachment with 1 μm RI for 3D culture (Figure 4 and 5).

### 2.6 Improved Cardiac Differentiation Capacity of hiPSC Spheroids Produced via CrMPD

To further validate the significantly improved quality of the hiPSC spheroids produced by crMPD without RI supplementation for 3D culture, compared to Versene detachment and supplementation with 10 μm RI for 3D culture, differentiation of the hiPSC spheroids into beating cardiac organoids was conducted by using a commercially available PSC cardiomyocyte differentiation kit. Cardiomyocyte differentiation was chosen because the quality of differentiation could be visually assessed and conveniently quantified, as functional cardiac organoids exhibit strong beating when viewed under a microscope. Although strong beating is observable for cardiac organoids produced from both groups after 15 days of differentiation (Movie S3, Supporting Information), the crMPD group has a significantly higher percentage of beating organoids starting from day 9 of differentiation than the group of Versene detachment and 10 μm RI supplementation (Figure 6D). This high cardiac differentiation efficiency in the crMPD group is further supported by the high expression of cardiac markers cTNI and α‐actinin with both qualitative immunofluorescence microscopy (Figure 6E) and quantitative flow cytometry (Figure 6F: 95.1 ± 2.6% and 94.3 ± 2.6% for cTNI and α‐actinin, respectively) analyses. These data confirm that hiPSC spheroids produced via crMPD without RI supplementation for 3D culture have greater differentiation potential than the hiPSC spheroids grown from the conventional way using Versene for detachment and supplementation of 10 μm RI for 3D culture.

## 3 Discussion

In this work, the crMPD (Figure 1 and 2, S1, Supporting Information), a device for convenient and mass production of high‐quality hiPSC spheroids with narrow size distribution (Figure 3A,B, 5, and S10–S11, Supporting Information), is developed using a facile microcontact printing method (Figure 1A). The specific pNIPAAm‐BA copolymer chosen for making the cold‐responsive surface has an LCST of 4–8 °C, which means that cells can only be released from the device upon cooling below that temperature. The LCST is much lower than room and cell‐incubation temperatures (≈22 and 37 °C), so that cells are not accidentally released during routine media changes and viewing under a microscope at room temperature. Although other studies have investigated the use of pathway inhibitors to prevent spontaneous differentiation of hiPSCs for use in high‐volume suspension culture bioreactors,^[^ ^49^ ^]^ these methods can't control the size of hiPSC colonies for producing homogeneous 3D spheroids, and the use of bioreactors can be laborious and expensive due to both the equipment involved and the quantity of specialized media needed to culture cells under these conditions. In this study, the crMPD can be used with standard hiPSC culturing methods. Importantly, the crMPD enables the cold‐triggered release of homogeneous hiPSC colonies (Figure 1B, 3C,D, Movies S1–S2, 5, and S10–S11, Supporting Information), which is an easily acceptable method that does not require any specialized reagents or equipment except a fridge or ice maker that is usually available in biomedical research labs and clinical facilities. Additionally, the release of cells at this ice‐cold temperature may help to slow cell metabolism and oxygen demand,^[^ ^50^ ^]^ which could have a beneficial effect on the cells and lessen the possible adverse impact of conventional detachment methods that usually involve incubation at 37 °C for a few minutes.^[^ ^51^ ^]^ While other methods have been developed to form homogenous high‐quality hiPSC spheroids in the absence of RI, such as through microfluidic tools,^[^ ^25^ ^]^ these methods also often require significant technical expertise to operate and can be laborious and expensive to set up and utilize. This differs from the crMPD technology that is relatively low cost, time‐efficient, and requires minimal technical proficiency.

A PDMS‐stamp‐based microcontact printing approach was used here to pattern the ECM on the cold‐responsive surface for cell attachment. The cell attachment matrix used here is Matrigel, but other proteins, such as vitronectin, collagen, and laminin,^[^ ^52^, ^53^ ^]^ could be used for more defined cell culture conditions. Indeed, our data show recombinant vitronectin is a suitable ECM protein for our micropatterning technology to fabricate crMPDs for hiPSC culture (Figure S2, Supporting Information). The microcontact printing approach is easily modifiable, and the design of the stamp can be altered to create different sizes, shapes, and patterns of ECM for transferring onto the cold‐responsive surface. The PDMS stamp can also be easily reused many times after simple cleaning and sterilization in 70% aqueous ethanol solution.

The design in this study used circular Matrigel islands of 100 and 400 μm in diameter for cell attachment, but the diameter of these islands could be modified by creating a new PDMS stamp design. The micropattern of the crMPD can be tuned to produce hiPSC colonies of different sizes for different differentiation applications. For example, one study found that hiPSC spheroids 500–600 μm in diameter are optimal for hepatic differentiation.^[^ ^54^ ^]^ The crMPD can therefore be customized to produce hiPSC spheroids of the desired size for the desired differentiation protocol. Because the crMPD could be designed with specific hiPSC colony sizes (400 μm in this case), the hiPSCs only needed to be cultured in 3D suspension for 2 days after release from the crMPD to self‐assemble and grow into the desired size. This results in spheroids with a narrow size distribution (Figure 5, S10, and S11, Supporting Information). However, the hiPSC colonies detached via Versene did not have such a method to control the initial size, and the initial colonies produced by this method varied greatly in size, much more so than the hiPSC colonies detached from the crMPD. Because the size of the initial colonies is difficult to control with the Versene detachment, the final hiPSC spheroids varied greatly in size, with a very wide size distribution (Figure 5, S10, and S11, Supporting Information), which is not ideal for differentiation applications. The crMPD can be customized to design patterns for producing hiPSC spheroids of the desired size, shortening the time needed to self‐assemble in suspension culture and presumably reducing uncontrolled fusion/merging of cell aggregates during prolonged suspension culture.^[^ ^55^ ^]^

The hiPSC spheroids produced via the crMPD show high viability and yield (Figure 4), a narrow size distribution (Figure 5, S10, and S11, Supporting Information), and high expression of pluripotency markers (Figure 6A–C). The hiPSC spheroids produced via the crMPD were compared to hiPSC spheroids produced via a conventional method for generating hiPSC spheroids: detachment of 2D hiPSCs using Versene and subsequent suspension culture of the detached hiPSC colonies until they assemble into hiPSC spheroids, helped by the addition of RI to the medium. RI works to prevent anoikis‐induced apoptosis that may occur during detachment/dissociation into single hiPSCs associated with Versene‐based detachment. Increasing the amount of RI in the media during 3D suspension culture of the Versene‐detached hiPSCs indeed helps the cells to survive, as evidenced by the higher viability and yield of the 10 μm RI group compared to the 1 μm RI group (Figure 4 and S3–S8, Supporting Information). However, the higher concentration of RI shows unwanted effects on hiPSC pluripotency, as the 10 μm RI group shows a trend of significantly lower expression of two pluripotency markers compared to the 1 μm RI group (Figure 6C), which has also been shown before.^[^ ^14^ ^]^ Because the hiPSC colonies released from the crMPD are not dissociated into single cells and presumably should maintain ECM and cell–cell connections in each colony from 2D culture, they are able to form into high‐quality hiPSC spheroids without the need for RI with high viability and significantly higher yield (Figure 4C, S3–S8, Supporting Information) and cardiac differentiation capacity (Movie S3, Supporting Information) than the 10 μm RI Versene‐detached hiPSC spheroids and at least comparable quality to the 1 μm RI Versene‐detached hiPSC spheroids in terms of pluripotency marker expression (Figure 6C). In other words, the crMPD method combines all the advantages of the Versene‐detachment methods with 1 and 10 μm RI while eliminating their drawbacks.

## 4 Conclusions

In summary, a cold‐responsive, micropatterned device (crMPD) is developed for cell culture to generate high‐quality and homogeneous hiPSC spheroids. The hiPSCs can successfully attach and grow on the micropatterned cell attachment design that is microprinted onto the cold‐responsive pNIPAAm‐BA‐coated surface. The hiPSC growth is confined only to areas of micropatterned Matrigel so that defined hiPSC colonies of a specific size are generated. These hiPSC colonies can be easily released from the crMPD surface by cooling the device on ice (0 °C), which is below the LCST of the pNIPAAm‐BA (4–8 °C). Under 3D suspension culture, the released hiPSC colonies self‐assemble into 3D hiPSC spheroids with high viability, yield, and pluripotency without the need for any RI supplementation in the culture medium. Because the hiPSC colonies are patterned to be the same size on the surface of the crMPD, the resulting hiPSC spheroids have a narrow size distribution, ideal for downstream differentiation of the hiPSC spheroids into the desired cell lineages. This method can be used for convenient mass production of homogeneous, high‐quality hiPSC spheroids for lineage‐specific differentiation, which is invaluable for facilitating the advance of hiPSC‐based research and personalized medicine.

## 5 Experimental Section

5.1

5.1.1

##### Synthesis of pNIPAAm‐BA

The pNIPAAm‐BA with an LCST range of 4–8 °C was synthesized by controlling the polymerization of NIPAAm (ThermoFisher Scientific, Waltham, MA, USA) and BA (Sigma‐Aldrich, Rockville, MD, USA) using a previously reported procedure.^[^ ^56^, ^57^ ^]^ Briefly, the molar ratio of NIPAAm to BA was set at 83:17, where 16.6 mmol NIPAAm and 3.4 mmol BA were added to the reaction flask in 10 mL of dioxane (Sigma‐Aldrich). After purging with nitrogen gas for 30 min, 0.5% w/v 2,2‐Azobis(2‐methylpropionitrile) (AIBN, Sigma‐Aldrich) in 5 mL of dioxane was added dropwise into the reaction flask. The reaction flask was then heated to 70 °C and stirred at 400 rpm under nitrogen for 12 h. The polymerization was stopped by exposing the flask to air and cooling down to room temperature (≈22 °C). Afterward, the resultant sample was precipitated in excess ice‐cold diethyl ether (Fisher Scientific, Waltham, MA, USA) and filtered through a filter paper (Diameter: 12.5 cm, VWR, Radnor, PA, USA). After dissolving in tetrahydrofuran (Fisher Scientific) (≈10 mL) and precipitating in ice‐cold diethyl ether three times, the synthesized polymer was dried overnight under a vacuum. The synthesized pNIPAAm‐BA has a number average molecular weight (M_n_) of 78 000 Da, polydispersity index (PDI) of 1.6, and molar ratio (x:y, X for NIPAAm and y for BA) of 5.8 ± 0.1.^[^ ^57^ ^]^

##### Coating of pNIPAAm‐BA on the Cell Culture Surface in Petri Dish

To create a pNIPAAm‐BA coating on the petri dish, a 5% w/v solution of pNIPAAm‐BA in ethanol was prepared. A total of 200 μL (by default) of the solution was dispensed onto a 35 mm Nunc Cell Culture/Petri Dishes (product #: 153 066, ThermoFisher Scientific) for spin coating. The two‐step procedure for spin‐coating the dish was first, spinning for 15 s at 150 RPM with an acceleration of 100 RPM and second, spinning for 30 s at 2000 RPM with an acceleration of 300 RPM. The pNIPAAm‐BA‐coated dishes were fully dried in a biosafety cabinet and sterilized by ultraviolet (UV) light for 20 min before use.

##### SEM and EDXS

For SEM imaging, dishes partially coated with pNIPAAm‐BA were cut into small pieces of 1 cm^2^ and attached to the SEM sample mount. The samples were coated with 10 nm thick Au/Pd using a sputter coater (CCU‐010 HV, Safematic, Zizers, Switzerland). Afterward, the SEM imaging and EDXS mapping and line scanning of the samples were conducted with a combined SEM and EDXS system (SU‐70 FEG, Hitachi, Tokyo, Japan) at 10 kV.

##### Contact Angle Measurement

For measuring contact angle, a goniometer (L2004A1, Ossila BV, Leiden, Netherlands) was used to take images of water droplets (10 μL) on uncoated cell culture dishes and the pNIPAAm‐BA‐coated dishes. The images were analyzed using ImageJ (v1.52q, NIH, USA).

##### Making the PDMS Stamp for Micropatterning of ECM

The design of micropatterning stamps (both 100 and 400 μm in diameter) was created using AutoCAD (Autodesk, Mill Valley, CA, USA), and a mask of the design was printed by CAD/Art Services Inc. (Brandon, OR, USA). To create the mold, SU‐82 050 photoresist (Kayaku Advanced Materials Inc., Westborough, MA, USA) was spin‐coated onto a silicon wafer (University Wafer, South Boston, MA) and soft baked on hot plates at 65 and 95 °C for 5 and 20 min, respectively. The wafer was then exposed to UV light through the patterned mask using an MA‐4 Mask Aligner (Karl Suss, Munich, Germany). Next, the wafer was hard baked at 65 and 95 °C for 5 and 10 min, respectively, and developed using SU‐8 developer (Kayaku Advanced Materials, Inc.) to expose the patterned features of crosslinked SU‐8. The spin speeds, baking parameters, and exposure energy were all determined based on the SU‐82 000 series datasheet from the manufacturer. The stamp was then made via soft lithography using this mold. The SYLGARD 184 Silicone Encapsulant Clear polydimethylsiloxane (PDMS, Dow, Midland, MI, USA) prepolymer and its curing agent (Dow) were mixed at a 10:1 (w/w, prepolymer:curing agent) and poured onto the mold. The PDMS on the mold was degassed in a vacuum and baked in a 75 °C oven for 2 h to cure/crosslink the polymer. The PDMS stamp was carefully cut and peeled out of the mold and subsequently sanitized in 70% ethanol for 30 min by sonication. The PDMS stamp was reused after a minimum of 1 h of cleaning via sonication and subsequent sterilization in 70% ethanol.

##### Micropatterning Cold‐Responsive Surface with PDMS Stamp

PDMS stamps were first thoroughly dried with nitrogen gas. The PDMS stamps were then plasma‐treated using a PDC‐32 G plasma cleaner (Harrick Plasma, Ithaca, NY, USA) for 3 min in sterile conditions. PDMS stamps were inked with Matrigel (Corning, Corning, NY, USA) diluted in dulbecco's modified eagle medium/nutrient mixture F‐12 (DMEM‐F12, Gibco, Gaithersburg, MD, USA) (20 μg mL^−1^) or Vitronectin XF (STEMCELL Technologies, Vancouver, BC, Canada) diluted in CellAdhere Dilution Buffer (STEMCELL Technologies, Vancouver, BC, Canada) (83 μg mL^−1^) and kept in biosafety cabinet on ice or at room temperature, respectively. The inking process for the PDMS stamp can be seen in Figure 1A. After 15 min of inking, the excess solution was aspirated, and the stamp was washed with deionized water to remove excess Matrigel or vitronectin not adsorbed onto the PDMS stamp surface. Nitrogen gas was blown onto the PDMS stamp to remove the excess liquid before being used for printing. Meanwhile, the pNIPAAm‐BA‐coated dish was plasma‐treated for 5 min in sterile conditions. Then, the inked PDMS stamp was placed feature‐side down on the pNIPAAm‐BA‐coated dish, and a 50 g weight with the same area as the PDMS stamp was placed on top to ensure full contact between the inked features on the stamp and the pNIPAAm‐BA‐coated dish. After 30 min of incubation at 37 °C and 5% CO_2_, the dish was ready for seeding cells. To better visualize the ECM pattern on the pNIPAAm‐BA‐coated surface, a mixture of Rhodamine B (Sigma‐Aldrich, 1 μg mL^‐^) and Matrigel (20 μg mL^−1^) was used to ink the PDMS stamps, and the same procedure was carried out to pattern the Rhodamine B/Matrigel mixture on the pNIPAAm‐BA‐coated surface. Images of the micropatterned surface were taken using a fluorescence microscope to visualize the pattern.

##### Cell Culture

This study used three different hiPSC cell lines. DF19‐9‐11 T.H (reprogrammed by a nonviral approach) and IMR90‐1 (reprogrammed by lentivirus infection) were purchased from WiCell (Madison, WI, USA). An additional hiPSC cell line was derived from HPF (PCS‐201‐102) from ATCC (Manassas, VA, USA) using the nonviral ReproRNA‐OKSGM Kit (STEMCELL Technologies, Vancouver, BC, Canada) by following the manufacturer's instructions. Briefly, HPF below passage five was cultured in a medium containing Dulbecco's Modified Eagle Medium (DMEM, Gibco) supplemented with 10% fetal bovine serum (FBS, Sigma‐Aldrich), 1 mм l‐glutamine (Invitrogen, Carlsbad, CA), and 1% MEM nonessential amino acids (NEAA, Thermofisher Scientific). HPF were then trypsinized and seeded overnight on a Matrigel‐coated 6‐well plate at 100 000 cells per well. HPF were then switched to a growth medium containing Advanced DMEM (Gibco), supplemented with 10% FBS, 1 mм l‐glutamine, and recombinant B18R protein (STEMCELL Technologies) at 175 ng mL^−1^; and incubated for 30 min. Afterward, HPF were transfected with a cocktail of RNA encoding hiPSC transcription factors (ReproRNA, STEMCELL Technologies) according to the manufacturer's protocol. Transfected HPF were left in the growth medium containing the transfection cocktail overnight and then selected by culturing the cells in growth medium containing 0.8 μg mL^−1^ puromycin (STEMCELL Technologies) for 5 days. On day 6, cells were switched to growth medium without puromycin for 2 days. On day 8, cells were switched to ReproTESR (STEMCELL Technologies) medium containing 175 ng mL^−1^ of B18R protein for 7 days. On day 15, cells were switched to ReproTESR containing no B18R protein. On day 28, the cells were switched to StemFlex medium (Gibco) until visible colonies were formed. Colonies were then picked using a 20‐gauge needle and seeded on a Matrigel (Corning)‐coated 6‐well dish and cultured in StemFlex containing 10 μm RI (Selleck Chemicals, Houston, TX, USA) and incubated overnight.

For 2D culture, all three hiPSC cell lines were maintained in StemFlex medium on Matrigel‐coated 6‐well plates (1 × 10^6^ cells in 1.5 mL of medium per well) at 37 °C and 5% CO_2_ with daily medium change. The cells were passaged twice a week at a ratio of 1:4 or 1:5 with Versene (ThermoFisher Scientific) detachment.

To obtain traditional 3D hiPSC spheroids (using Versene and RI), hiPSC colonies under 2D culture at 80% confluency were incubated with Versene for 4 min at 37 °C. Then, the 2D hiPSC colonies were gently washed twice with 1× phosphate‐buffered saline (PBS, Thermofisher Scientific) right after incubation to remove Versene. The colonies were then gently detached by flushing with fresh StemFlex medium. Afterward, the detached hiPSCs were filtered through a 400 μm cell strainer (Millipore Sigma, Rockville, MD, USA) as control of the 400 μm micropattern and resuspended in StemFlex with 0, 1, or 10 μm RI and 0.35% v/v methylcellulose (R&D Systems, Minneapolis, MN, USA). The cells were transferred into a 10 cm polystyrene petri dish for 2 days of suspension culture with RI. To generate hiPSC spheroids using the crMPD without RI, hiPSC colonies under 2D culture at 80% confluency were detached and dissociated using Versene. Cells were then gently washed twice with 1× PBS to remove Versene. The detached cells were then plated on the crMPD at 1 × 10^4^ cells cm^−2^ and allowed to attach overnight. Cells were cultured on the crMPD for 4 days with StemFlex medium, and the medium was changed every day. Confluent hiPSCs on the crMPD were then detached by cooling the dish on ice (≈0 °C) for ≈15 min. The detached hiPSCs were then collected via pipetting, transferred to a 5 mL centrifuge tube, allowed to sink to the bottom of the tube (≈5 min), and washed with precooled (on ice) StemFlex to remove any dissolved pNIPAAm‐BA in the solution. Cells were transferred to StemFlex supplemented with 0.35% methylcellulose and cultured in a polystyrene petri dish for 2 days in 3D suspension culture to allow for spheroid formation.

##### Cryomicroscopy for Cell and Matrigel Detachment on crMPD

The pNIPAAm‐BA was spin‐coated onto a quartz sample holder, and the ECM pattern was micropatterned onto it as described above. To visualize cell detachment, hiPSCs were seeded and allowed to attach overnight onto micropatterned Matrigel islands. After 2 days of growth for 100 μm crMPD and 4 days of growth for 400 μm crMPD, the quartz window was placed on the cryostage (Linkam Scientific, Surrey, UK) to allow for controlled cooling of the sample while imaging the cells. Precooled (in 4 °C) StemFlex medium was added to the stage. The sample was cooled from 10 to 0 °C at a rate of 1 °C min^−1^, then held at 0 °C for 10 min. Images were taken every other second during the holding time at 0 °C to visualize hiPSCs and ECM patterns detaching from the substrate.

##### Viability and Yield of Cells Detached Using Versene Versus crMPD

The viability of cells detached using Versene with 0, 1, or 10 μm RI or via cold treatment on the 400 μm Matrigel island in crMPD with 0 μm RI was evaluated by live/dead staining of the detached cells. After either 2 h or 48 h of 3D suspension culture postdetachment, the cells were stained with calcein AM (1 μm) and PI (1 μg mL^−1^) to visualize live (green stain) and dead (red stain) cells, respectively. Cells were incubated with the dye for 10 min at 37 °C and 5% CO_2_ before imaging with a Zeiss (Oberkochen, Germany) LSM710 microscope to capture green and red fluorescence. Cell viability was calculated as the percentage of the area of live cells/cell aggregate area out of the total area of cells/cell aggregates.

To determine the cell yield based on the detachment method, the same number of hiPSCs were detached from 2D culture using Versene and 1 or 10 μm RI or using cold treatment on the 400 μm Matrigel island in crMPD and cultured in 3D suspension for 2 days. Afterward, the cells that formed aggregates were collected by centrifugation at 300 RPM for 3 min. Cells were dissociated into single cells using 0.25% trypsin‐EDTA (ThermoFisher Scientific) and counted using a hemacytometer.

To study their size distribution and shape, the hiPSC spheroids generated from the different methods were imaged using a Zeiss LSM710 microscope. The outlines of the hiPSC spheroids were drawn, and the area (*A*) and perimeter (*P*) of each spheroid were quantified using the Zen Blue software (Oberkochen, Germany). The equivalent diameter (*D*) of each hiPSC spheroid was calculated as: $D=2\sqrt{A/\pi }$, and the circularity (*C*) was calculated as: $C=\left(4\pi A\right)/\left(P^{2}\right)$.

##### Flow Cytometry for Evaluating hiPSC Pluripotency Markers

For flow cytometry, the DF19‐9‐11 T.H hiPSC spheroids made by either Versene detachment with 1 or 10 μm RI supplementation or cold treatment on the 400 μm Matrigel island in crMPD with 0 μm RI supplementation were collected after 2 days of 3D suspension culture to create hiPSC spheroids. The spheroids were dissociated into single cells using 0.25% trypsin‐EDTA for 5 min at 37 °C. The cells were fixed with 4% paraformaldehyde (PFA, ThermoFisher Scientific) in 1× PBS for 10 min at RT, permeabilized with 0.1% Triton‐X (Sigma‐Aldrich) for 5 min, blocked for nonspecific binding using 3% bovine serum albumin (BSA, Sigma‐Aldrich) in 1× PBS for 1 h at RT, and incubated with primary antibodies (1:500 dilution using 3% BSA solution) overnight at 4 °C. The next day, cells were incubated with a secondary antibody (1:1000 dilution using 3% BSA) for 1 h at RT in the dark. The cells were washed with 1× PBS and analyzed using a BD Biosciences (San Jose, CA, USA) FACSCelesta flow cytometer. The primary antibodies were NANOG (CST #4893, Cell Signaling Technologies, Danvers, MA, USA), OCT4 (ab19857, Abcam, Cambridge, UK), SOX2 (ab93689, Abcam), and SSEA‐4 (CST #4755, Cell Signaling Technologies). The secondary antibodies were either goat anti‐mouse (A‐28 175, ThermoFisher Scientific) or goat anti‐rabbit Alexa Fluor 488‐tagged antibodies (A‐11 008, ThermoFisher Scientific). All flow data were analyzed using FlowJo v10 (BD Bioscience).

##### Cryosectioning and Immunostaining for Evaluating hiPSC Pluripotency Markers

The DF19‐9‐11 T.H hiPSC spheroids were fixed in 4% PFA in 1× PBS at 4 °C overnight. The fixed spheroids were transferred to 10% sucrose (Sigma‐Aldrich) in PBS for 4 h and then 15% sucrose in PBS for 4 h. Afterward, the spheroids were embedded in Optimal Cutting Temperature (OCT, Tissue‐Tek Sakura) compound for cryosection. The spheroids were sectioned into 10 μm thick slices using a Leica (Buffalo Grove, IL, USA) cryostat platform and then attached onto Leica Apex high‐adhesive glass slides. A standard immunostaining protocol was used to visualize pluripotency markers for the hiPSC spheroids. Slides were rinsed with 1× PBS to remove OCT and then incubated with 0.1% Triton X‐100 (Sigma‐Aldrich) to permeabilize the cells. Nonspecific binding was blocked using a 3% BSA solution in 1× PBS for 1 h at room temperature. Afterward, samples were incubated with primary antibodies at 4 °C overnight (1:500 dilution). The primary antibodies were OCT‐4, SSEA‐4, SOX2, and NANOG. Afterward, the samples were rinsed with 1× PBS and incubated with the associated secondary antibodies (goat anti‐rabbit IgG Alexa Fluor 488 and goat anti‐mouse IgG Alexa Fluor 568 (A‐11 004, ThermoFisher Scientific), 1:1000 dilution) in 1× PBS for 1 h at room temperature. The samples were then rinsed with 1× PBS and stained for 10 min with 1 μg mL^−1^ 4′,6‐diamidino‐2‐phenylindole (DAPI, Sigma‐Aldrich) to visualize the nuclei. Samples were imaged with a Zeiss LSM 710 microscope.

##### Cardiac Differentiation of 3D hiPSC Spheroids

To further analyze the pluripotency difference between 3D hiPSC spheroids from conventional 3D culture (with RI and Versene detachment) and crMPD, cardiac differentiation of the spheroids was studied by using a commercially available PSC cardiomyocyte differentiation kit (ThermoFisher Scientific), per the manufacturer's instructions. Briefly, the DF19‐9‐11 T.H hiPSC spheroids made by either Versene detachment with 10 μm RI supplementation or cold treatment on 400 μm Matrigel island in crMPD with 0 μm RI supplementation were collected after 2 days of 3D suspension culture. The hiPSC spheroids were transferred to cardiomyocyte differentiation medium A and cultured for 2 days. Then, the spheroids were transferred to cardiomyocyte differentiation medium B and cultured for 2 days. The resultant cardiac spheroids were transferred to cardiomyocyte maintenance medium and cultured for 11 days. The media were changed every other day. For immunofluorescence staining and flow cytometry, the cardiac spheroids differentiated from DF19‐9‐11 T.H hiPSC spheroids made by cold treatment on the 400 μm Matrigel island in crMPD with 0 μm RI supplementation were then either sectioned and prepared for staining or dissociated and prepared for flow cytometry using the same methods for pluripotent markers. The primary antibodies were cTNI (ab47003, Abcam, Cambridge, UK) and α‐actinin (A7732, Millipore Sigma, Rockville, MD, USA). The secondary antibodies were either goat anti‐mouse (A‐28 175, ThermoFisher Scientific) or goat anti‐rabbit Alexa Fluor 488‐tagged antibodies (A‐11 008, ThermoFisher Scientific). All flow data were analyzed using FlowJo v10 (BD Bioscience).

##### Statistical Analysis

Data were reported as the mean ± standard deviation from at least three independent experiments. For comparison between more than two groups, one‐way analysis of variance (ANOVA) followed by Tukey *post hoc* correction was used, assuming Gaussian distribution. The homogeneity of variance of one‐way ANOVA was verified using the Brown‐Forsythe test. Two‐tailed unpaired *t*‐test and two‐tailed paired *t*‐test, using the parametric test and assuming equal variance and Gaussian distribution, were performed for comparing two groups. Differences were considered statistically significant when the *p*‐value was <0.05. *p < 0.05, **p < 0.01, ***p < 0.001, and ****p < 0.0001, and ns: not significant. All graphing and statistical analyses were performed using GraphPad Prism 10 (GraphPad Software, San Diego, CA, USA).

## Conflict of Interest

X.H. filed patent application for the crMPD technology reported in this work through his startup company HOHCells, LLC (https://www.hohcells.com) with permission from the University of Maryland, College Park.

## Author Contributions

**Xiaoming He** conceived the project and supervised the study; **Xiaoming He** (leading), **Zhiyuan Wang** (supporting), and **Samantha Stewart** (supporting) designed experiments; **Zhiyuan Wang** and **Samantha Stewart** equally conducted experiments with assistance from **Mitsuo Kumagai**, **Wenquan Ou**, **Ethan Wang**, **Yongyu Lu**, **Taotao Meng**, and **Cancan Xu**; **Xiaoming He**, **Zhiyuan Wang**, **Samantha Stewart,** and **Mitsuo Kumagai** equally analyzed data; **Zhiyuan Wang** and **Samantha Stewart** equally wrote the manuscript draft and generated and edited figures; **Xiaoming He**, **Samantha Stewart**, **Zhiyuan Wang**, **Yi Hong**, **Shenqiang Ren**, and **Mitsuo Kumagai** equally edited the manuscript; and all authors approved the manuscript. **Zhiyuan Wang**, **Samantha Stewart**, and **Mitsuo Kumagai** contributed equally to this work.

## Supporting information

Supplementary Material

## Data Availability

The data that support the findings of this study are available from the corresponding author upon reasonable request.
