# Supplementary material for: High‐Yield Convenient Mass Production of High‐Quality Homogenous Human Induced Pluripotent Stem Cell Spheroids under Rho‐Associated Kinase Inhibitor‐Free 3D Culture Enabled by Micropatterning and Cold‐Triggered Chemical‐Free Cell Detachment
Source: Small Sci. 2025 Jul 11;5(9):2500201. doi: 10.1002/smsc.202500201 (PMC12412540; doi:10.1002/smsc.202500201)
Supplement: Supplementary file 1 — Supplementary Material [file SMSC-5-2500201-s001.zip › smsc202500201-sup-0001-SuppData-S1.pdf]

Supporting Information (Figures S1-S13 and Captions of Movies S1-S3)

High-Yield Convenient Mass Production of High-Quality Homogenous Human Induced Pluripotent Stem Cell Spheroids Under Rho-Associated Kinase Inhibitor-Free 3D Culture Enabled by Micropatterning and Cold-Triggered Chemical-Free Cell Detachment

*Zhiyuan Wang[#], Samantha Stewart[#], Mitsuo Kumagai[#], Ethan Wang, Wenquan Ou, Yongyu Lu, Taotao Meng, Cancan Xu, Yi Hong, Shenqiang Ren, and Xiaoming He**

[#]These authors contributed equally

*Correspondence should be addressed to Xiaoming He (shawnhe@umd.edu)

Supplementary Figures

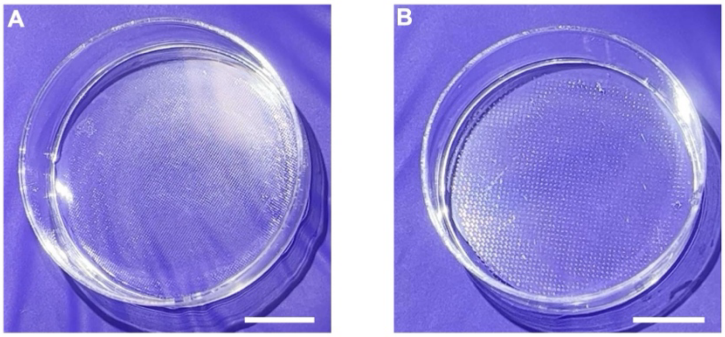

Figure S1. Photos of crMPDs with Matrigel micropatterns of different sizes. A, 100 µm micropatterns. **B**, 400 µm micropatterns. The micropatterns can be seen as the many tiny white dots in the photos. Approximately 2,000 micropatterns of 400 µm and 15,000 micropatterns of 100 µm can be made in a 35 mm dish. Scale bars: 1 mm.

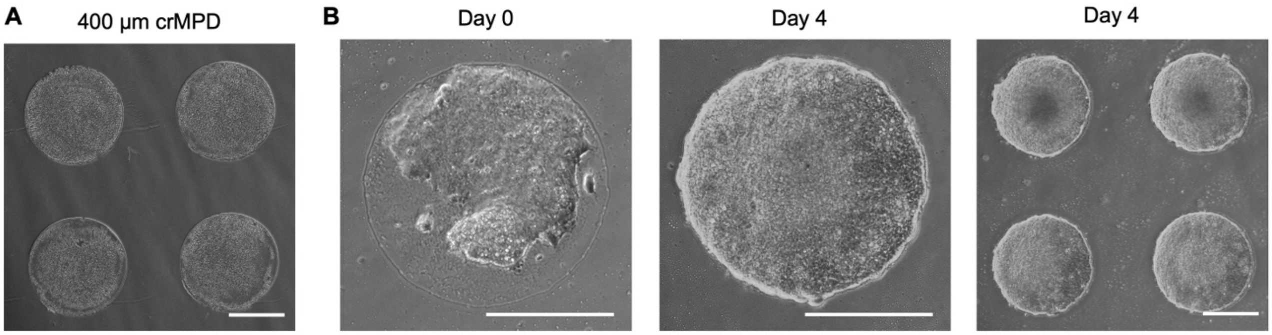

Figure S2. Representative phase images of hiPSCs cultured in the vitronectin crMPD. **A,** Vitronectin islands (400 μm) micropatterned on top of the cold-responsive pNIPAM-BA layer in the crMPD. The vitronectin islands are uniform and round. Scale bar: 100 μm. **B,** DF19-9-11T.H hiPSC attachment on the 400 μm vitronectin islands/micropatterns in crMPD on different days. The hiPSCs could attach and grow in 4 days to form well defined colonies with clear boundary on the vitronectin islands. Scale bars: 200 μm (left and middle) and 100 μm (right).

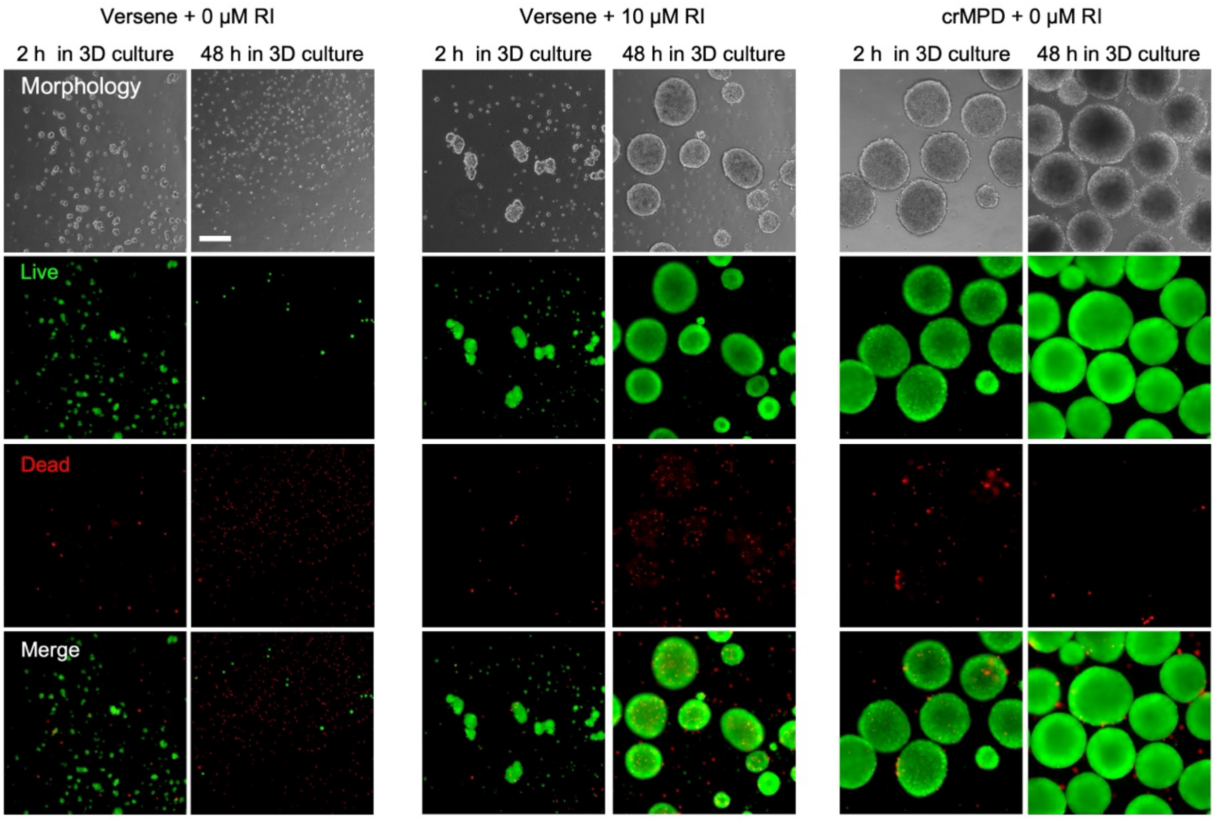

Figure S3. Live and Dead Staining of IMR90-1 hiPSCs detached and 3D-cultured in three different ways. Representative images of the viability assessed by live/dead staining of hiPSCs at 2 and 48 h post-detachment (PD) either using Versene and supplementation with 0, 10 µM RI for 3D culture or by cold treatment from crMPD with 400 µm diameter Matrigel islands and supplementation with 0 µM RI for 3D culture. Scale bar: 200 µm.

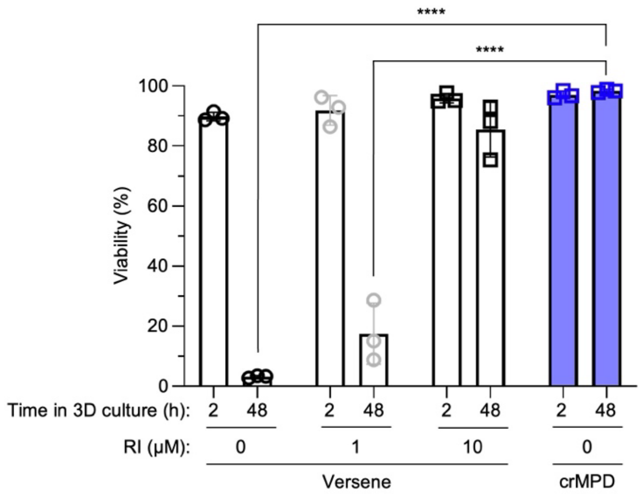

Figure S4. Quantitative data on the viability of IMR90-1 hiPSCs detached and 3D-cultured in four different ways. The viability is assessed by live/dead staining of hiPSCs at 2 and 48 h post-detachment (PD) either using Versene and supplementation with 0, 1, 10 µM RI for 3D culture or by cold treatment from crMPD with 400 µm diameter Matrigel islands and supplementation with 0 µM RI for 3D culture. Statistical analyses were done using one-way analysis of variance (ANOVA) with Tukey's multiple comparisons test and correction. ****$p < 0.0001$. n = 3 independent runs. Error bars represent standard deviation.

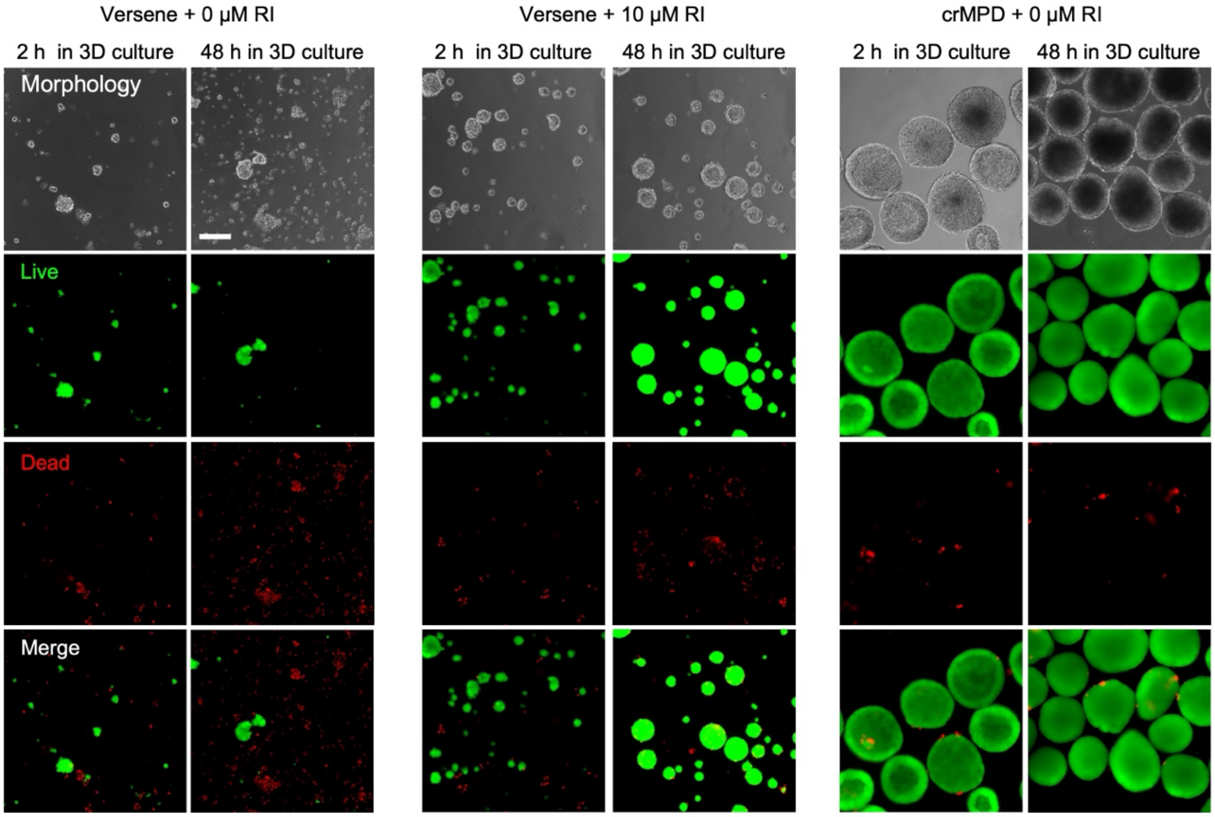

Figure S5. Live and Dead staining of reprogrammed hiPSCs detached and 3D-cultured in three different ways. Representative images of the viability assessed by live/dead staining of hiPSCs at 2 and 48 h post-detachment (PD) either using Versene and supplementation with 0, 10 μM RI for 3D culture or by cold treatment from crMPD with 400 μm diameter Matrigel islands and supplementation with 0 μM RI for 3D culture. Scale bar: 200 μm.

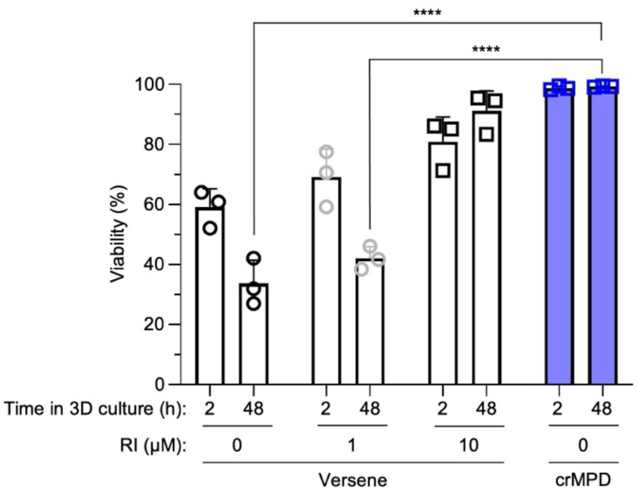

Figure S6. Quantitative data on the viability of reprogrammed hiPSCs detached and 3D-cultured in four different ways. The viability is assessed by live/dead staining of hiPSCs at 2 and 48 h post-detachment (PD) either using Versene and supplementation with 0, 1, 10 µM RI for 3D culture or by ice cold treatment from crMPD with 400 µm diameter Matrigel islands and supplementation with 0 µM RI for 3D culture. Statistical analyses were done using one-way analysis of variance (ANOVA) with Tukey's multiple comparisons test and correction. ****$p < 0.0001$. n = 3 independent runs. Error bars represent standard deviation.

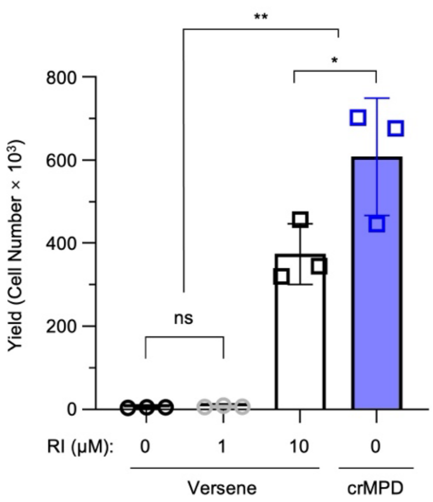

Figure S7. Yield of IMR90-1 hiPSCs detached and 3D-cultured in four different ways. The cell yield is assessed at 48 h post detachment either using Versene and supplementation with 0, 1, 10 µM RI for 3D culture or by ice cold treatment from crMPD with 400 µm diameter Matrigel islands and supplementation with 0 µM RI for 3D culture. Statistical analyses were done using one-way analysis of variance (ANOVA) with Tukey's multiple comparisons test and correction. *$p < 0.05$, **$p < 0.01$, and ns: not significant. n = 3 independent runs. Error bars represent standard deviation.

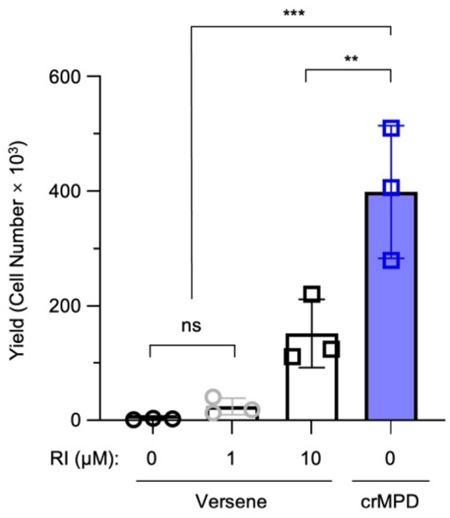

Figure S8. Yield of reprogrammed hiPSCs detached and 3D-cultured in four different ways. The cell yield is assessed at 48 h post detachment either using Versene and supplementation with 0, 1, 10 µM RI for 3D culture or by ice cold treatment from crMPD with 400 µm diameter Matrigel islands and supplementation with 0 µM RI for 3D culture. Statistical analyses were done using one-way analysis of variance (ANOVA) with Tukey's multiple comparisons test and correction. $^{**}p < 0.01$, $^{***}p < 0.001$ and ns: not significant. n = 3 independent runs. Error bars represent standard deviation.

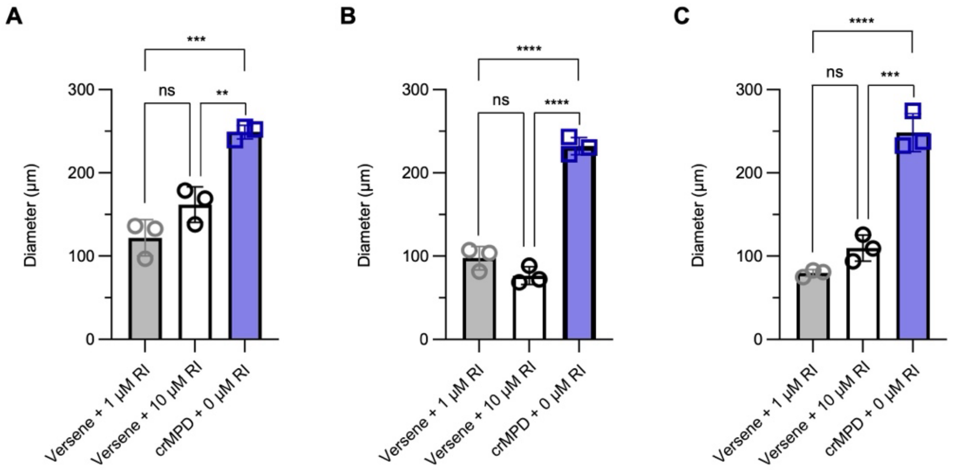

Figure S9. Average size of the hiPSC spheroids detached and 3D-cultured in three different ways. **A**, DF19-9-11T.H. hiPSCs **B**, IMR90-1 hiPSCs. **C**, Reprogrammed hiPSCs. Statistical analyses were done using one-way analysis of variance (ANOVA) with Tukey's multiple comparisons test and correction. **$p < 0.01$, ***$p < 0.001$, ****$p < 0.0001$, and ns: not significant. n = 3 independent runs. Error bars represent standard deviation.

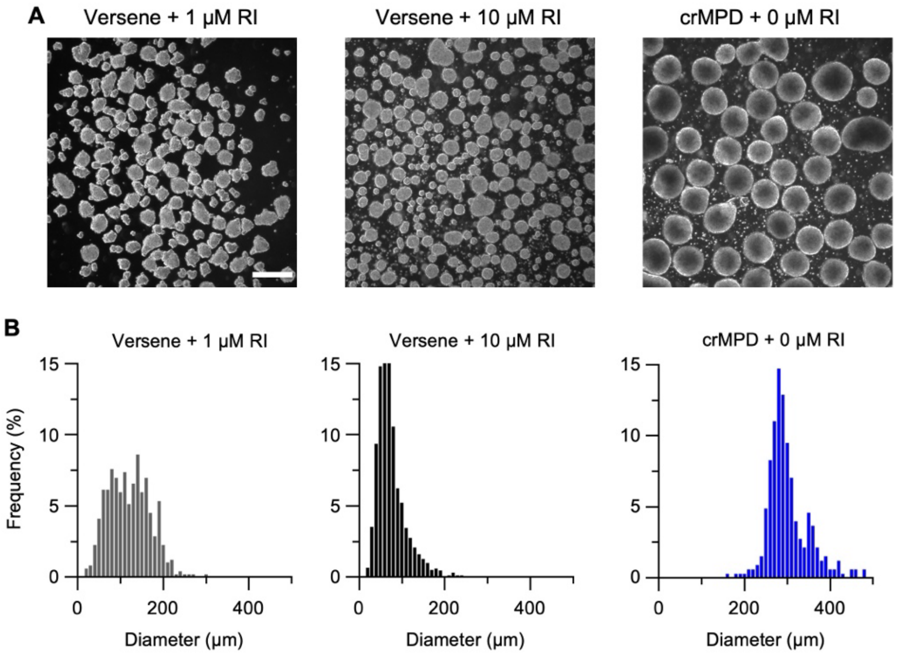

Figure S10. Homogeneous size of IMR90-1 hiPSC spheroids produced in three different ways. **A**, representative images, and **B**, histogram showing size distribution, of hiPSC spheroids produced after detachment either using either Versene supplemented with 1 or 10 µM RI for 3D culture (two conventional ways) or by ice cold treatment of the crMPD supplemented with 0 µM RI for 3D culture (crMPD-based approach). The IMR90-1 hiPSC spheroids produced by the cdMPD-based approach are more homogeneous and larger than that produced by the two conventional ways. n = 3 independent runs. Scale bar: 500 µm.

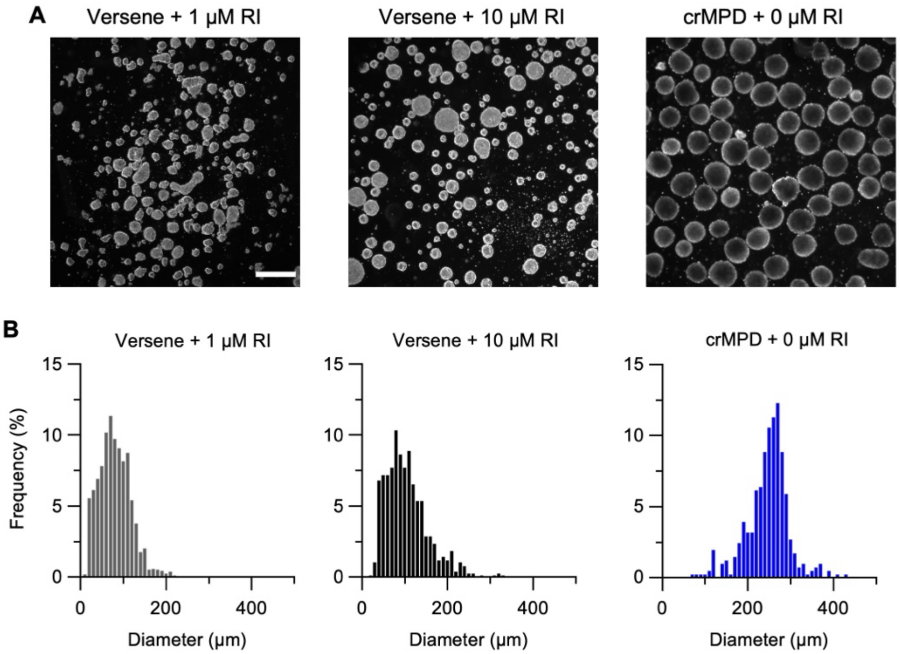

Figure S11. Homogeneous size of reprogrammed hiPSC spheroids produced in three different ways. A, representative images, and **B**, histogram showing size distribution, of hiPSC spheroids produced after detachment either using either Versene supplemented with 1 or 10 μM RI for 3D culture (two conventional ways) or by ice cold treatment of the crMPD supplemented with 0 μM RI for 3D culture (crMPD-based approach). The reprogrammed hiPSC spheroids produced by the crMPD-based approach are more homogeneous and larger than that produced by the two conventional ways. n = 3 independent runs. Scale bar: 500 μm.

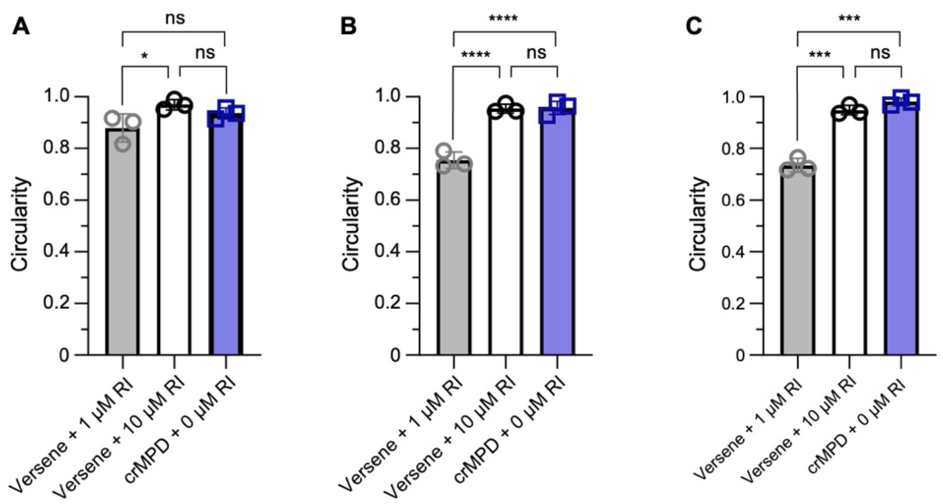

Figure S12. Circularity of hiPSC spheroids produced in three different ways. **A**, DF19-9-11T.H. hiPSCs. **B**, IMR90-1 hiPSCs. **C**, Reprogrammed hiPSCs. Statistical analyses were done using one-way analysis of variance (ANOVA) with Tukey's multiple comparisons test and correction. *$p < 0.05$, ***$p < 0.001$, ****$p < 0.0001$, and ns represents not significantly different. n = 3 independent runs. Error bars represent standard deviation.

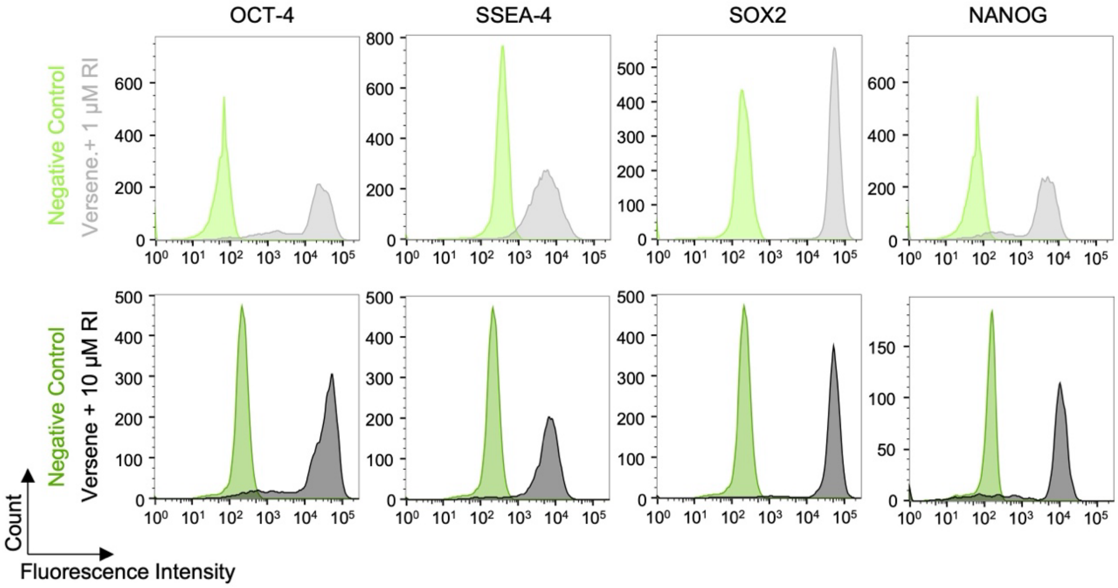

Figure S13. Representative flow cytometry peaks showing the expression the pluripotency markers in hiPSC spheroids produced by Versene detachment. The DF19-9-11T.H hiPSC spheroids detached by Versene supplemented with 1 or 10 μM RI for 3D culture are shown in the top and bottom rows, respectively. OCT-4, SSEA-4, SOX2, and NANOG are pluripotency markers. The negative controls (secondary antibody only, no primary antibody) are shown in light green (1 μм, top row), dark green (10 μм, bottom row).

Supplementary Movie Captions

Movie S1. Ice cold-triggered detachment of 2D hiPSC colony grown on 100 μm Matrigel micropatterns in crMPD. Scale bar: 50 μm.

Movie S2. Ice cold-triggered detachment of 2D hiPSC colony grown on 400 μm Matrigel micropatterns in crMPD. The detached colony in this movie does not move as much as the detached colony in Movie S1, because the former is much (~4 times in diameter and 16 times in area) bigger than the latter. Scale bar: 100 μm.

Movie S3. Beating cardiac organoids (on day 15) differentiated from hiPSC spheroids grown from hiPSCs cold-detached from crMPD for 3D culture without any RI. Scale bar: 200 μm.
